# CD44 Participates to Extramedullary Haematopoiesis Onset by Mediating the Interplay Between Monocytes and Haematopoietic Stem Cells in Myelofibrosis

**DOI:** 10.1111/jcmm.70720

**Published:** 2025-07-21

**Authors:** Margherita Mirabile, Camilla Tombari, Anita Neroni, Lara Tavernari, Ruggiero Norfo, Elisa Bianchi, Monica Maccaferri, Barbara Mora, Sandra Parenti, Chiara Carretta, Matteo Bertesi, Marica Malerba, Elisa Papa, Luca Fabbiani, Niccolò Bartalucci, Paola Guglielmelli, Leonardo Potenza, Lorena Losi, Francesco Passamonti, Enrico Tagliafico, Mario Luppi, Sebastiano Rontauroli, Alessandro Maria Vannucchi, Rossella Manfredini, the MYNERVA (Myeloid NEoplasms Research Venture AIRC) investigators

**Affiliations:** ^1^ Interdepartmental Centre for Stem Cells and Regenerative Medicine University of Modena and Reggio Emilia Modena Italy; ^2^ Department of Biomedical, Metabolic and Neural Sciences University of Modena and Reggio Emilia Modena Italy; ^3^ Department of Life Sciences University of Modena and Reggio Emilia Modena Italy; ^4^ Hematology Unit, Department Oncology and Hematology Modena University Hospital Modena Italy; ^5^ Ospedale Maggiore Policlinico Milan Italy; ^6^ Pathology Unit, Department of Medical and Surgical Sciences University of Modena and Reggio Emilia Modena Italy; ^7^ CRIMM, Center Research and Innovation of Myeloproliferative Neoplasms University of Florence, AOU Careggi Florence Italy; ^8^ Department of Experimental and Clinical Medicine University of Florence Florence Italy; ^9^ Department of Medical and Surgical Sciences University of Modena and Reggio Emilia Modena Italy; ^10^ University of Milan Milan Italy; ^11^ Diagnostic Hematology and Clinical Genomics Unit, Department of Laboratory Medicine Modena University Hospital Modena Italy

**Keywords:** CD44, cell migration, extramedullary haematopoiesis, hyaluronic acid, myelofibrosis, osteopontin

## Abstract

Extramedullary haematopoiesis (EMH) refers to blood generation outside of the bone marrow (BM). In Myelofibrosis (MF), a myeloproliferative neoplasm, the disruption of BM microenvironment promotes haematopoietic stem and progenitor cells (HSPCs) mobilisation, resulting in the onset of EMH in the spleen, and then in splenomegaly. Although JAK2 inhibitors have a good efficacy in reducing splenomegaly, the presence of a significant proportion of non‐responder patients underlines the need to explore the cellular mechanisms responsible for the EMH onset. In a MF mouse model, Ruxolitinib induces a reduction in spleen volume but does not affect EMH. CD44 inhibition successfully reduces monocyte and HSPC migration in an in vitro extravasation model. Strikingly, MF monocytes are more effective in promoting HSPC migration through the production of hyaluronic acid. Collectively, our results demonstrate that CD44 regulates the migration of monocytes that are crucial for the onset of EMH in MF patients, as they produce CD44 ligands recruiting HSPCs from the BM.

## 1 Introduction

Extramedullary haematopoiesis (EMH) refers to the onset of the blood cell generation process in organs and anatomical regions distinct from the bone marrow (BM) which is the site of haematopoiesis in healthy individuals. EMH occurs as a response to haematopoietic stress that can be induced by various conditions, such as infections, malignancies, anaemia, and metabolic stress that compromise the proper functioning of the BM microenvironment [1]. The common sites of malignant EMH include the spleen, liver, and lymph nodes [2]. While the frequency of circulating haematopoietic stem and progenitor cells (HSPCs) in the peripheral blood (PB) of healthy individuals is very low [3], changes in the BM microenvironment promote the release of HSPCs from the BM into PB in a process called mobilisation. The mobilised HSPCs that settle in peripheral organs are responsible for the onset of EMH [4]. Because of the continuous expansion of haematopoietic cells, EMH can cause organomegaly and a series of complications, including the compression of the surrounding structures or the rupture and bleeding of the swollen organs themselves.

Myeloproliferative neoplasms (MPNs) are a family of clonal haematological disorders originating from HSPCs and characterised by the hyperproliferation of myeloid precursors, which leads to the overproduction of differentiated cells [5]. Philadelphia‐negative MPNs include primary myelofibrosis (PMF), polycythaemia vera (PV) and essential thrombocythaemia (ET) that can evolve to secondary myelofibrosis (MF) [6, 7]. In MF patients, many HSPCs are released from the BM into PB due to the progressive onset of BM fibrosis and the extensive production of cytokines and soluble factors. The migration of these mobilised HSPCs is promoted by the increased expression of stromal cell‐derived factor 1 (SDF1) [8] and is the basis for EMH development, in particular in the spleen, where HSPCs find a supporting microenvironment due to the increased expression of haematopoietic cytokines by activated splenic vascular endothelial cells [9, 10].

Stem cell‐derived clonal myeloproliferation in MF is elicited by driver mutations affecting *JAK2*, *CALR*, or *MPL* genes [11]. These driver mutations sustain clonal myeloproliferation through the constitutive activation of the JAK/STAT signalling pathway downstream of thrombopoietin receptor [12]. JAK inhibitors, such as Ruxolitinib and Fedratinib, have been developed to target this pathway and have proved to be effective in providing a substantial clinical benefit by alleviating symptoms and inducing spleen size reduction (≥ 35%) in MF patients. This reduction can be attributed to the depletion of a subpopulation of MF progenitors but not of stem clones [13]. Moreover, substantial spleen response cannot be achieved in a sizable fraction of patients receiving JAK inhibitors [10, 14] and splenomegaly remains an unmet clinical need. New pharmacological approaches are under investigation to avoid the discomfort and the anatomical complications caused by splenomegaly and the eventual splenectomy or splenic irradiation [15]. However, the clinical scenario remains challenging and, since EMH represents the systemic spreading of the neoplastic clone and a reservoir for MF propagating stem cells [16], contrasting EMH might be a promising strategy to interfere with disease progression.

A major hallmark of MF is the increase in pro‐inflammatory cytokines plasma levels that creates an altered microenvironment that participates in the maintenance of the neoplastic clone [17]. Among pro‐inflammatory and pro‐fibrotic mediators, we previously demonstrated that Osteopontin (OPN) plasma levels are increased in PMF compared to ET and PV patients and correlate with a more severe BM fibrosis degree and a shorter overall survival [18]. OPN promotes mesenchymal stromal cell growth and collagen I production in vitro. Moreover, we successfully inhibited OPN production with the ERK1/2 inhibitor Ulixertinib in a MF mouse model induced by the administration of a thrombopoietin receptor agonist (TPO‐RA), Romiplostim. Ulixertinib reduced BM fibrosis deposition in mice either alone or in combination with Ruxolitinib, and this effect can be mainly ascribed to the reduced OPN production since it could be recapitulated through the administration of anti‐OPN neutralising antibody [19]. Despite its pro‐fibrotic role, it is well known that OPN can promote cell migration and in particular accumulation and persistence of macrophages [20]. Moreover, OPN is a key molecule in regulating homing, retention, and function of HSPCs [21] as the OPN^−/−^ mouse shows an aberrant distribution of HSPCs in the BM [22].

Starting from the observation that in a MF mouse model Ruxolitinib reduces splenomegaly but did not restore spleen histology, we investigated the molecular mechanisms underlying EMH development and the role played by monocytes in HSPCs migration. Based on the high OPN plasma level in MF mice and patients, we focused on its chemoattractive role and investigated OPN receptors inhibition as a putative strategy to counteract monocytes and HSPCs migration. Thanks to an in vitro extravasation model based on Transwell membrane coated with endothelial cells, we deepened the molecular mediators of monocytes and HSPCs migration, highlighting the primary contribution of CD44 surface protein.

## 2 Materials and Methods

### 2.1 Patients

The study was conducted using human HSPCs, monocytes, and plasma samples collected from PB of healthy donors (HDs) and MF patients, which were diagnosed according to 2022 World Health Organisation criteria [6, 7] (Table S1). The study was performed in accordance with the Declaration of Helsinki and was approved by the local ethical committee (Comitato Etico dell'Area Vasta Emilia Nord, AVEN). All subjects provided informed written consent. PB samples from HDs were collected from Azienda Ospedaliero‐Universitaria (AOU) Policlinico di Modena, University of Modena and Reggio Emilia, according to the institutional guidelines for discarded material.

### 2.2 Mice

Animal studies were reviewed and approved by the Italian Ministry of Health. The MF mouse model was established as previously described using only female animals [19, 23]. Mice were sacrificed on day 15, spleens were harvested, and spleen index was calculated as spleen weight/body weight ×100. Spleens were fixed and paraffin‐embedded. Organ sections were stained according to the haematoxylin and eosin protocol (Bio‐Optica, Milan, Italy) or subjected to immunohistochemical analysis using antibodies against murine F4/80 (ThermoFisher cat. #14‐4801‐82, Waltham, Massachussets, USA) by means of the Ventana BenchMark Discovery XT (Roche, Swiss). The images were captured by using an Axioscope A1 microscope equipped with an AxioCam ERc 5S Digital Camera and Axion software 4.8 (all Carl Zeiss MicroImaging Inc.; Thornwood, NY, USA). Megakaryocyte count was performed with ImageJ (Rasband, W.S., National Institutes of Health, USA), with the plugin Cell_Counter. F4/80 signal quantification was performed with ImageJ with the plugin Colour_Deconvolution2, applying the H‐DAB vector and then measuring the percentage of stained area.

### 2.3 Antibodies and Drugs for Migration Experiments

Immunophenotyping was performed on PBMCs from HDs and MF patients and cryopreserved cells from mice spleens as previously described [24] (for the list of antibodies and gating strategies see Supporting Information). Data were analysed with FlowJo v10.8 Software (BD Life Sciences, New Jersey, USA).

For in vitro extravasation assay the following antibodies and corresponding isotype controls were used: monoclonal anti‐CD44 antibody (clone IM7, cat. #14‐0441‐82), rat IgG2a kappa Isotype Control as control IgG for the monoclonal anti‐CD44 antibody (clone eBR2a, cat. #14‐4321‐82; both from eBioscience, Invitrogen, ThermoFisher, Waltham, Massachussets, USA); Polyclonal anti‐CD44 antibody (clone HCAM DF1485, cat. #sc‐7297), anti‐αvβ3 antibody (clone 23C6, cat. #sc‐7312), normal mouse IgG as control IgG for the polyclonal anti‐CD44 and anti‐αvβ3 antibodies (cat. #sc‐2025, all from Santa Cruz Biotechnology Inc., Dallas, Texas, USA); anti‐α4β1 antibody, *InVivoMAb* anti‐mouse/human VLA‐4 (CD49d) (clone PS/2, cat. #BE0071), *InVivoMAb* rat IgG2b isotype control as control IgG for the anti‐α4β1 antibody (clone LTF‐2, cat. #BE0090, all from BioXCell, Lebanon, New Hampshire, USA); anti‐human OPN Antibody (cat. AF1433, Biotechne, R&D systems, Minnesota, USA); Hyaluronan inhibitor (cat. #AS‐62622, Anaspec, California, USA).

We used also Ruxolitinib (INCB18424, cat# HY‐50856, MedChemExpress, Monmouth Junction, NJ, USA), resuspended in dimethyl sulfoxide (DMSO, Sigma‐Aldrich, Merck, Darmstadt, Germany).

### 2.4 In vitro Migration Assay

Migration experiments were performed using the Transwell system (Corning, NY, USA). Experimental conditions were defined according to preliminary experiments as detailed in Supporting Information. To obtain Transwell membrane coating with endothelial cells, 80,000 HUVEC cells were seeded on the upside down Transwell insert, treated with 100 μg/mL TNF‐α (Miltenyi Biotech, Bergisch Gladbach, Germany) and cultured overnight. Then, CD14^+^ monocytes and CD34^+^ HSPCs were immunomagnetically isolated from PBMCs using the corresponding MicroBead Kits UltraPure (Miltenyi Biotech, Bergisch Gladbach, Germany) as previously described [19, 25] and detailed in Supporting Information. 800,000 CD14^+^ monocytes or 100,000 CD34^+^ HSPCs were loaded on Transwell with or without coating. After overnight migration, cells were collected from the lower chamber and counted with CountBright Plus Absolute Counting Beads (C36995, ThermoFisher, Waltham, Massachussets, USA). For CD14^+^ cells, no specific chemoattractant was added in the lower chamber because FBS was sufficient to induce cellular migration; SDF‐1 (Miltenyi Biotech, Bergisch Gladbach, Germany) 200 ng/mL served as a chemoattractant for CD34^+^ cells.

### 2.5 Statistical Analysis

Results of flow cytometry‐based counting of migrated cells were represented in each graph as normalised cell count. A correction factor was obtained by dividing not treated (NT) sample cell count for each experiment by NT samples' mean cell count. Every treated sample was then divided by this correction factor to reduce the high standard deviation among the replicates.

Values were reported as mean ± standard deviation (SD); the normal distribution of the data was verified by the Shapiro–Wilk test. Statistical analysis was performed using the paired *T*‐test for the analysis of migration experiments and the unpaired *T*‐test or one‐way ANOVA for other comparisons based on the number of experimental groups. *p*‐values were considered statistically significant when < 0.05. Statistical analysis was performed using GraphPad Prism version 9.5.1 for macOS (GraphPad Software, Boston, Massachusetts USA, www.graphpad.com). Images were created with BioRender.com.

## 3 Results

### 3.1 Ruxolitinib Reduces Splenomegaly but Does Not Abrogate EMH in MF Mice

**FIGURE 1:**
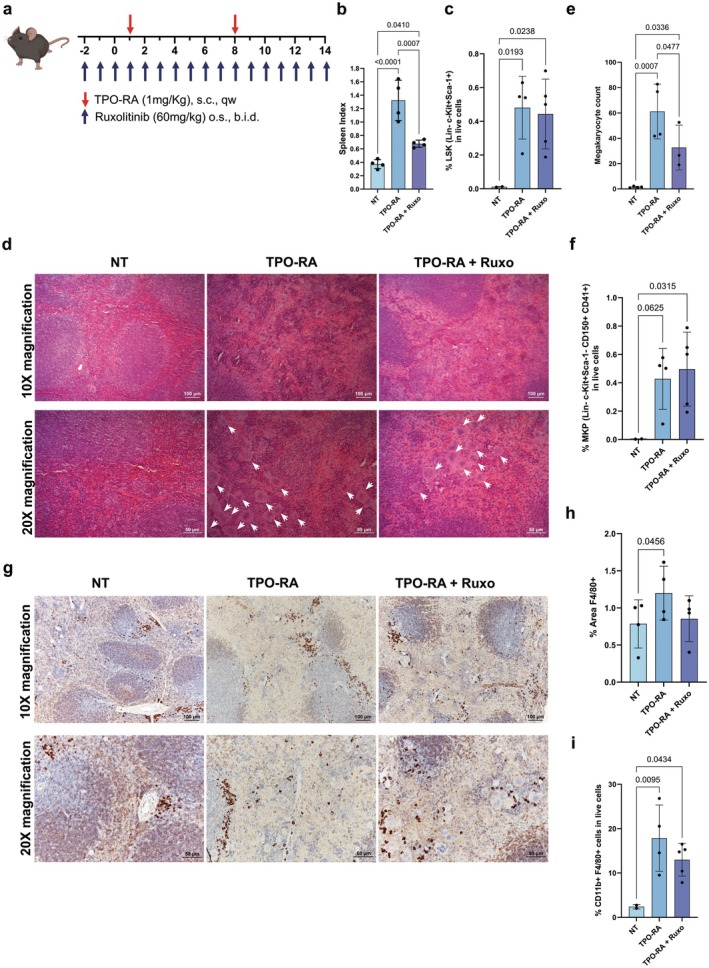
EMH takes place in the spleen of TPO‐RA mice. (a) Schematic outline for the generation of MF mice. MF was induced by sub‐cutaneous injection of a TPO‐RA (Romiplostim, 1 mg/kg, once weekly). Mice were given Ruxolitinib 60 mg/kg through oral gavage twice daily starting 3 days before the first TPO‐RA injection. Mice were sacrificed after 15 days of TPO‐RA treatment. (b) Spleen index at sacrifice of not treated (NT), TPO‐RA treated and TPO‐RA + Ruxolitinib treated mice (*n* = 4 for each group). (c) Flow cytometry evaluation of Lineage^−^ Sca1^+^ and c‐Kit^+^ (LSK) cells in the spleen of NT (*n* = 2), TPO‐RA (*n* = 4) and TPO‐RA + Ruxolitinib (*n* = 5) treated mice. Each dot represents a mouse. (d) Representative images of haematoxylin–eosin stained spleen sections from controls, TPO‐RA treated mice and MF mice receiving Ruxolitinib. Megakaryocytes are indicated using white arrows in 20X images and represent large cells with multilobed nuclei and abundant cytoplasm. (e) The megakaryocyte count for NT (*n* = 4), TPO‐RA treated (*n* = 4) and TPO‐RA + Ruxolitinib treated mice (*n* = 3). Each dot represents the mean count of megakaryocytes in at least 3 fields of the same section at 10X magnification (*n* = 4 for each group). (f) Flow cytometry evaluation of Lineage^−^ Sca1^−^ c‐Kit^+^ CD150^+^ CD41^+^ megakaryocyte progenitors in the spleen of NT (*n* = 2), TPO‐RA (*n* = 4) and TPO‐RA + Ruxolitinib (*n* = 5) treated mice. Each dot represents a mouse. (g) Immunohistochemistry for F4/80 of NT, TPO‐RA treated and TPO‐RA and Ruxolitinib treated mice. (h) Immunohistochemistry quantification of spleen sections from NT, TPO‐RA treated and TPO‐RA and Ruxolitinib treated mice. Each dot represents the percentage of area covered by F4/80 signal in a representative field (*n* = 4 for each group). (i) Flow cytometry evaluation of CD11b^+^ F4/80^+^ macrophages in the spleen of NT (*n* = 2), TPO‐RA (*n* = 4) and TPO‐RA + Ruxolitinib (*n* = 5) treated mice. Each dot represents a mouse. In graphs each dot represents a mouse, each column represents group mean ± standard deviation. Comparisons were performed by means of one‐way ANOVA. *p*‐values are reported only if < 0.05. Scale bars of 100 and 50 μm are shown for 10× and 20× magnification images, respectively.

To understand the molecular basis of EMH, we exploited a MF mouse model obtained through the administration of a TPO‐RA, Romiplostim (Nplate) [19, 23] (Figure 1a), which develops a sizable splenomegaly in 14 days (Figure 1b, Figure S1). Spleen sections from TPO‐RA treated mice (TPO‐RA mice) display profound changes in stroma architecture. While spleens from untreated (NT) mice display clearly distinct lobular and round white pulp regions surrounded by red pulp with red blood cells and F4/80^+^ macrophages, in TPO‐RA treated mice the white pulp disappears, or is strongly reduced forming small compact lymphatic nodules, and the spleen is colonised by haematopoietic cells invading red pulp regions (Figure S1). In this MF model, Ruxolitinib treatment causes a decrease in splenomegaly (Figure 1b, Figure S1A), as happens in MF patients, although the normal spleen size and architecture is not fully recovered (Figure S1).

The presence of Lineage^−^Sca1^+^c‐Kit^+^ (LSK) HSPCs, megakaryocyte precursors and megakaryocytes in spleens from TPO‐RA mice and their absence in NT animals represents a distinctive and measurable sign of EMH (Figure 1c–f, Figure S2). Interestingly, Ruxolitinib only partially reduces spleen megakaryocyte count without affecting the frequency of LSK cells and megakaryocyte precursors (Figure 1c–f) and does not restore the normal spleen architecture (Figure S1), thus indicating that EMH is not fully inhibited.

We evaluated macrophage number in the spleen of TPO‐RA mice by means of immunohistochemical analysis and flow cytometry using F4/80 as a macrophage‐specific marker. As shown in Figure 1g–i, F4/80^+^ macrophages are increased in TPO‐RA mice, suggesting that splenomegaly is associated with their buildup in the spleen. Therefore, Ruxolitinib treatment induces only a partial, non‐significant reduction in the number of macrophages infiltrating the spleen that does not impact EMH extension (Figure 1h,i).

Based on such evidence, we hypothesised that monocytes, migrated to the spleen, differentiate into macrophages and act as primers for EMH [26, 27, 28] by producing chemoattractive molecules for HSPCs, which undergo extravasation and constitute the EMH islands in the spleen.

### 3.2 Ruxolitinib Does Not Affect Monocyte and HSPC Migration in vitro

**FIGURE 2:**
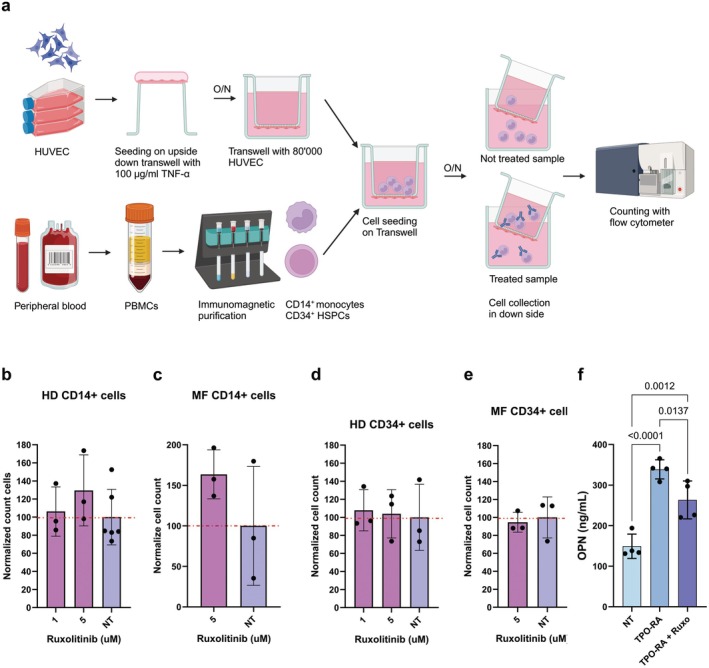
The effect of Ruxolitinib on splenomegaly is not mediated by cell migration but it is correlated with OPN. Scheme in panel (a) represents the workflow of our in vitro extravasation system based on Transwell with HUVECs. Each Transwell received 80,000 HUVEC cells treated with 100 μg/mL of TNF‐α. HUVECs were seeded upside down for an overnight incubation. The next day the Transwell modified with HUVECs was loaded with immunopurified CD14^+^ or CD34^+^ cells. After an overnight incubation, the cells in the down side of the system were collected and counted with flow cytometry. Panels (b–e) show the effect of Ruxolitinib treatment on in vitro migration of, respectively, HD CD14^+^ cells (*n* = 3), MF CD14^+^ cells (*n* = 3), HD CD34^+^ cells (*n* = 3) and MF CD34^+^ cells (*n* = 3). The red dashed line represents 100% migration. On *X* axis are reported Ruxolitinib doses, on *Y* axis the normalised cell count (see Section 2). (f) The OPN plasma levels for NT, TPO‐RA treated and TPO‐RA + Ruxolitinib treated mice. In graphs each dot represents a replicate for migration experiments or a mouse for ELISA assay, each column represents group mean ± standard deviation. Comparisons in mice were performed by means of one‐way ANOVA for panels (b, d, f), and unpaired *T*‐test for panels (c, e). *p*‐values are reported only when < 0.05.

Given these results, we looked for molecular pathways that could be targeted to counteract EMH onset through the inhibition of HSPC and monocyte extravasation. To investigate which molecules could affect the migration of human monocytes and HSPCs, we set up a simple and reproducible in vitro extravasation model as detailed in Supporting Information: Results and Figure S3. Briefly, to mimic the extravasation process, Transwell inserts were covered with HUVEC cells activated with TNF‐α to induce the expression of adhesion molecules typical of an inflamed endothelium, like what happens in MF patients [29]. PB‐derived CD14^+^ monocytes or CD34^+^ HSPCs were loaded in the upper chamber of each well, and migrated cells were counted after overnight incubation (Figure 2a).

We evaluated Ruxolitinib's effect in this in vitro extravasation model and we found that treatment does not inhibit migration of HD and MF monocytes but, on the contrary, it slightly induces it (Figure 2b,c). Moreover, Ruxolitinib does not impact cell migration of HD and MF HSPCs at any tested concentration (Figure 2d,e).

Because Ruxolitinib does not affect cell viability (Figure S6) and migration in vitro, we hypothesised that the slight reduction in macrophage number observed in MF mice spleens could involve other mechanisms, for example, inhibition of inflammatory cytokines. This hypothesis was supported by the reduction of OPN plasma level in TPO‐RA mice that was observed after Ruxolitinib treatment (Figure 2f). Since OPN is a well‐known chemoattractant molecule for monocytes [30], we investigated the role of OPN in promoting monocyte and HSPC migration in MF.

### 3.3 OPN Receptors Expression Is Deregulated in MF Cells

**FIGURE 3:**
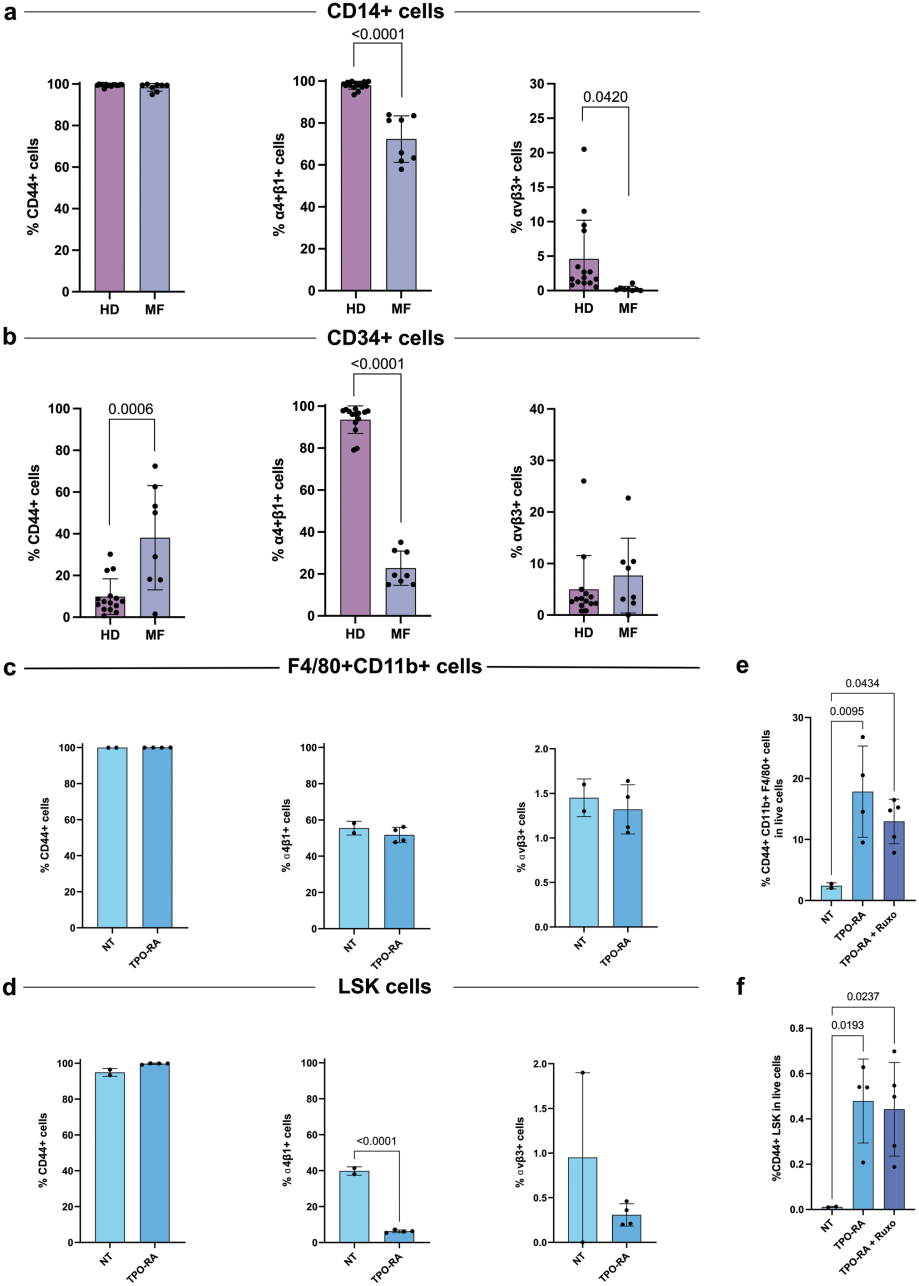
CD44 is highly expressed in MF monocytes and HSPCs and its expression is increased in the spleen of MF mice. (a) Immunophenotypic evaluation of α4β1, αvβ3 and CD44 in circulating CD14 ^+^ monocytes in HDs (*n* = 15) and MF patients (*n* = 8). (b) Immunophenotypic evaluation of α4β1, αvβ3 and CD44 in circulating CD34 ^+^ cells in HDs (*n* = 15) and MF patients (*n* = 8). (c, d) Flow cytometry evaluation of CD44, α4β1 and αvβ3 expression in macrophages (c) and LSK cells (d) in the spleen of NT (*n* = 2) and TPO‐RA (*n* = 4) treated mice. Each dot represents a mouse. (e, f) Flow cytometry evaluation of the frequency of CD44 ^+^ macrophages (e) and LSK cells (f) in the spleen of NT (*n* = 2), TPO‐RA (*n* = 4) and TPO‐RA + Ruxolitinib (*n* = 5) treated mice. Each dot represents a mouse. In graphs, each dot represents a patient or a mouse, and each column represents group mean ± standard deviation. Comparisons were performed by means of unpaired *T*‐test for panels (a–d) and one‐way ANOVA for panels (e, f). *p*‐values are reported only when < 0.05.

To focus on the role of OPN in EMH onset in MF, we analysed the expression of its receptors already described as involved in cell migration. We evaluated by flow cytometry the expression of the OPN receptors CD44, αvβ3 and α4β1 integrins [31, 32, 33] in circulating HSPCs and monocytes from 8 MF patients and 15 HDs (Figures S4 and S5). Results show that 100% of monocytes express CD44 in both HD and MF samples (Figure 3a). The frequency of CD14^+^ cells expressing α4β1 and αvβ3 is reduced in MF samples compared with HD ones (Figure 3a).

As regards circulating HSPCs, CD44^+^ cells are three‐fold increased in MF patients as compared to HDs (38.1% ± 24.99% vs. 9.85% ± 8.54%, *p* = 0.006). On the contrary, we did not observe any difference in αvβ3^+^ cell frequency, whereas a strong reduction of α4^+^β1^+^ cells in MF patients was detected if compared to HDs (22.74% ± 8.17% vs. 93.56% ± 6.54%, *p* < 0.0001) (Figure 3b).

These results suggest that CD44 might play a pivotal role in regulating the capacity of MF monocytes and HSPCs to enter circulation and invade organs far from BM. This hypothesis is supported by the cytofluorimetric evaluation of OPN receptors expression in macrophages and LSK cells isolated from the spleen of TPO‐RA treated mice. As shown in Figure 3c,d in both compartments, αv^+^β3^+^ cells are rare while the frequency of α4^+^β1^+^ cells is not altered by TPO‐RA treatment among macrophages but is strongly reduced within LSK cells. On the other hand, CD44 is expressed by the totality of macrophages and LSK cells in both NT animals and TPO‐RA mice. Consequently, TPO‐RA treatment led to a significant increase in the frequency of CD44^+^ macrophages and LSK cells, and Ruxolitinib treatment only slightly reduces CD44^+^ macrophages (Figure 3e,f) supporting the hypothesis that the effect of JAK inhibition on EMH is limited.

### 3.4 Monoclonal Anti‐CD44 Antibody Strongly Inhibits HD and MF Monocyte Migration

Based on immunophenotypic analysis results and considering monocytes as primers for EMH, we first evaluated whether inhibition of OPN receptors might be able to interfere with HD monocyte migration in the established in vitro extravasation system. To this end, we tested the following inhibitory antibodies: anti‐CD44, monoclonal and polyclonal, anti‐α4β1 and anti‐αvβ3. No cytotoxic effect is elicited by any antibody at any concentration on HD monocytes (Supporting Information: Results, Figure S6) therefore excluding any nonspecific effect on cell migration.

**FIGURE 4:**
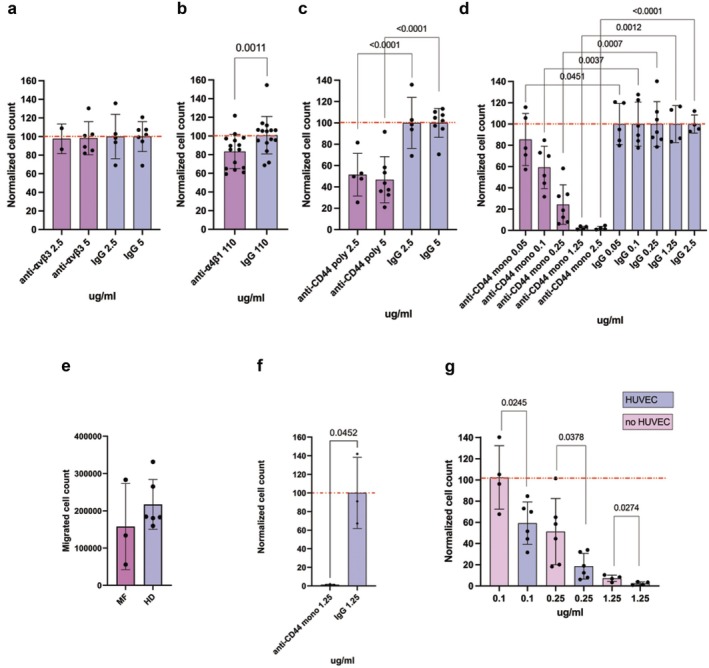
CD44 regulates HD and MF CD14^+^ monocyte migration. Panel (a) shows the effect of anti‐αvβ3 antibody on migration of HD CD14^+^ monocyte in vitro in presence of HUVEC coating while panel (b) displays results for the anti‐α4β1 antibody treatment. Panel (c) shows the effect of the polyclonal anti‐CD44 antibody on migration of HD CD14^+^ monocyte in vitro in presence of HUVEC endothelium while panel (d) displays results for the monoclonal anti‐CD44 antibodies. (e) Graph displays the difference in migration between HD (*n* = 6) and MF (*n* = 3) CD14^+^ monocytes in the established in vitro extravasation model. (f) The effect of anti‐CD44 antibody on migration of MF CD14^+^ monocyte in vitro (*n* = 3). (g) Bar plot represents results of HD CD14^+^ monocyte migration assays with and without HUVEC for the monoclonal anti‐CD44 antibody; Transwell samples with HUVEC are represented with purple bars while Transwell samples without HUVEC are illustrated with pink bars; the percentage of migration is normalised considering control IgG samples, not shown in this graph. In all bar plots the red dashed line represents 100% migration. On *X* axis are reported antibodies doses, *Y* axis reports the normalised cell count (see Section 2). In graphs each dot represents a replicate, each column represents group mean ± standard deviation. Comparisons between samples treated with the specific antibody or control IgG were performed by means of paired *T*‐test. *p*‐values are reported only if < 0.05.

According to ⍺vβ3 low expression, anti‐⍺vβ3 antibody does not affect monocyte migration (Figure 4a). On the contrary, anti‐⍺4β1 antibody induces a slight but statistically significant reduction in monocyte migration compared with control (Figure 4b). Anti‐CD44 antibodies are the most effective in reducing migration of HD monocytes in vitro: polyclonal antibody causes a 50% reduction in cell migration (Figure 4c), while anti‐CD44 monoclonal antibody causes a dose‐dependent reduction in monocyte migration that is completely inhibited at the two highest doses (Figure 4d).

Collectively, our results suggest that the CD44 adhesion molecule is the main responsible for HD monocyte migration compared with ⍺4β1 and ⍺vβ3 integrins. We also demonstrated that HD and MF monocytes migrate with a comparable efficacy through the Transwell membrane and that consistently CD44 inhibition is effective in ceasing MF monocyte migration (Figure 4e,f).

In the established in vitro migration model, the Transwell membrane was coated with HUVEC cells to mimic the extravasation of cells through vessel endothelium. To investigate how the presence of HUVEC cells influenced the effect of anti‐CD44 and anti‐⍺4β1 treatment on monocyte migration, we performed in vitro migration experiments with and without the HUVEC layer. We observed no differences in the effects elicited by the anti‐⍺4β1 antibody due to the presence or absence of HUVEC cells (Figure S7). On the contrary, when CD14^+^ monocytes are treated with monoclonal anti‐CD44 antibody, the reduction in migration is always greater when the endothelium is present (Figure 4g). As a result, the presence of activated HUVEC cells halves the IC50 value for monoclonal anti‐CD44 antibody (0.106 μg/mL with HUVEC coating vs. 0.194 μg/mL without HUVEC coating).

These results show that the presence of activated HUVEC cells potentiates the migration inhibition effect of anti‐CD44 antibody, supporting the idea that this antibody inhibits not only the extravasation of monocytes but also their recall by the inflamed endothelium.

### 3.5 Both OPN and HA Promote Migration of Monocytes in vitro

**FIGURE 5:**
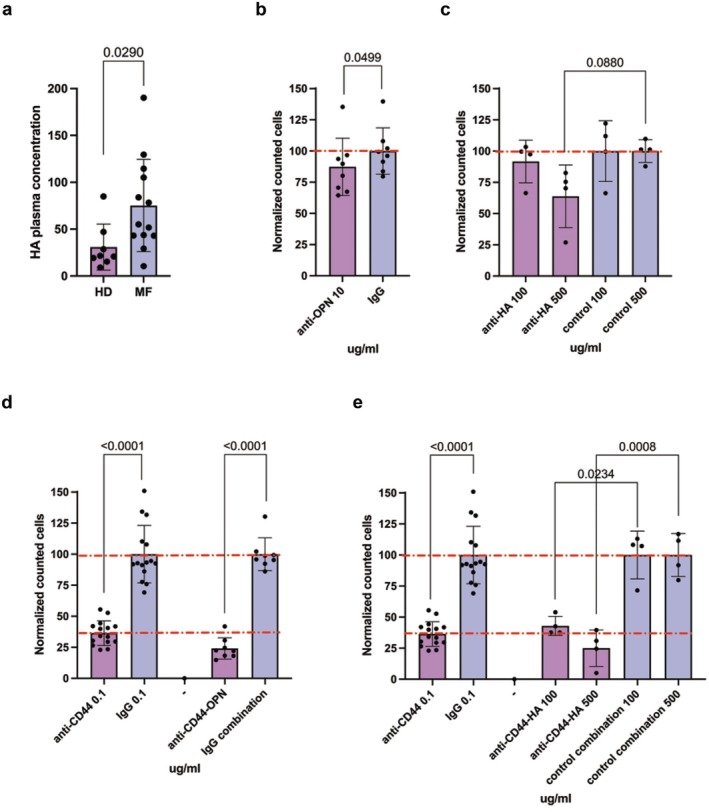
CD44 regulates CD14^+^ monocytes migration through the interaction with OPN and HA. (a) The graph displays the concentration of HA (ng/mL) in HD (*n* = 8) and MF (*n* = 13) plasma samples. Comparison between HD and MF was performed by means of unpaired *T*‐test. Panels (b, c) show, respectively, the effect of neutralising antibody against OPN and HA inhibitor on HD CD14^+^ monocyte migration. Panels (d, e) show the effect on HD CD14^+^ monocytes migration of the combination of neutralising antibody against OPN (d) or HA inhibitor (e) with the monoclonal anti‐CD44 antibody. The upper red dashed line represents 100% migration while the down red dashed line represents the migration inhibition induced by the anti‐CD44 0.1 μg/mL treatment. *X* axis display antibodies and inhibitor doses, *Y* axis shows the normalised cell count (see Section 2). In graphs each dot represents a replicate, each column represents group mean ± standard deviation. Comparisons between samples treated with the specific antibody or control IgG were performed by means of paired *T*‐test. *p*‐values are reported only if < 0.05.

We have previously demonstrated that OPN plasma concentration is significantly increased in MF patients as compared to HDs [18]. Moreover, OPN is a well‐known ligand for CD44 that promotes cell migration [26]. Nevertheless, CD44 ligands also include hyaluronic acid (HA) which might act as a chemoattractive molecule. To investigate the involvement of HA in regulating haematopoietic cell migration in MF, we evaluated HA plasma concentration in 13 MF patients and 8 HDs. Our analysis revealed that HA concentration is significantly increased in MF plasma samples: 75.20 ± 49.16 ng/mL in MF versus 30.80 ± 24.58 ng/mL in HD, *p* = 0.029 (Figure 5a).

Based on these observations, we tested the effect of an anti‐OPN antibody and a hyaluronan inhibitor in our in vitro extravasation system to verify which ligand was primarily involved in regulating monocyte migration. Both OPN and HA inhibition cause a slight reduction in monocyte migration in vitro (Figure 5b,c). However, we observed that the addition of anti‐OPN antibody or HA inhibitor little strengthens the effect of anti‐CD44 antibody (Figure 5d,e) suggesting that both ligands participate in regulating monocyte migration through the interaction with their common receptor.

### 3.6 MF HSPCs Are More Sensitive to CD44 Inhibition Than HD HSPCs

Given that the monoclonal anti‐CD44 antibody displayed the strongest effect in inhibiting HD monocyte migration, we evaluated its capacity to influence HSPC migration in vitro. We hypothesised that CD44 might be primarily involved in MF HSPC extravasation and migration due to its significant upregulation in MF CD34^+^ cells compared with HDs. To test this hypothesis, we performed in vitro extravasation experiments using circulating HSPCs isolated from both HDs' and MF patients' PB.

**FIGURE 6:**
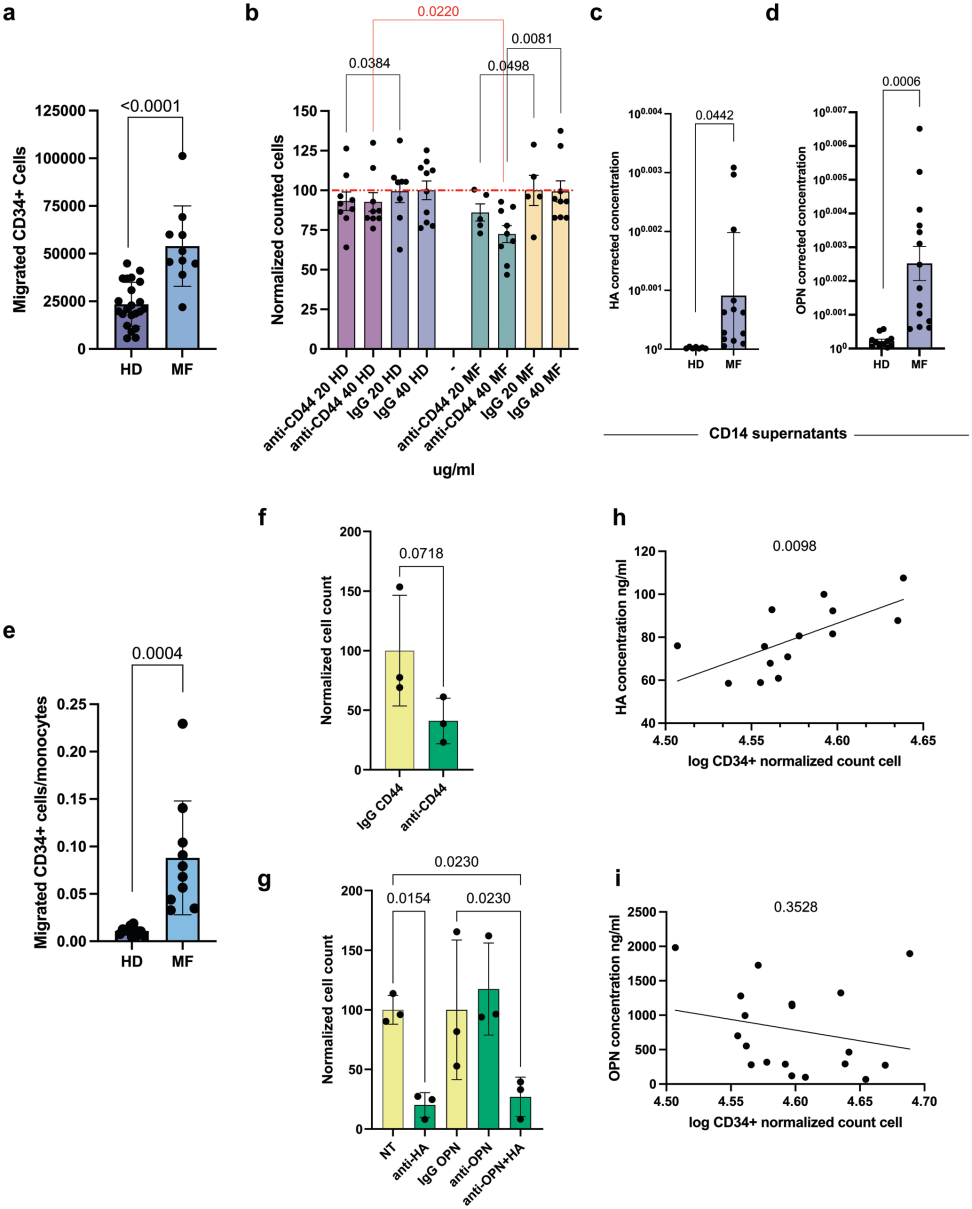
CD44 regulates CD34^+^ HSPC migration in vitro through the binding with HA. (a) Graph displays the difference in migration between HD (*n* = 22) and MF (*n* = 10) CD34^+^HSPCs in our in vitro extravasation model. (b) The plot shows the inhibition of migration induced by the anti‐CD44 monoclonal antibody on HD (*n* = 22) and MF (*n* = 10) CD34^+^ HSPCs. The red dashed line represents 100% migration. On *X* axis are reported the antibodies at different doses, on *Y* axis the normalised cell count (see Section 2). Each dot represents a replicate. Panels (c, d) show the OPN and HA produced by HD (*n* = 11) and MF (*n* = 13 for panel c and *n* = 14 for panel d) CD14^+^ monocytes in vitro, respectively. The number of live monocytes was determined at time of medium collection, 96 h after plating, and it was used to normalise ligand concentration to obtain a measure of OPN and HA produced by single monocytes in culture. (e) The effect of HD (*n* = 11) and MF (*n* = 10) CD14^+^ supernatants on HD CD34^+^ cell migration; the number of migrated CD34^+^ cells was normalised based on the number of remaining monocytes at time of supernatants harvesting. Each dot represents a replicate. Panel (f) shows the effect of anti‐CD44 monoclonal antibody on HD CD34^+^ cells migration induced by MF monocytes conditioned medium (*n* = 3). (g) The effect of anti‐OPN antibody and HA inhibitor, alone or in combination, on HD CD34^+^ cells migration induced by MF monocytes conditioned medium (*n* = 3). Panels (h, i) show the correlation between log transformed CD34^+^ normalised migrated cells and HA (panel h, *n* = 14) and OPN (panel i, *n* = 19) concentration. In graphs each dot represents a replicate, each column represents group mean ± standard deviation. Comparisons were performed by means of unpaired *T*‐test for panels (a, c, d, e), paired *T*‐test for panels (b, f), and one‐way ANOVA for panel (g). Correlation analysis was performed by simple linear regression. *p*‐values are reported only if < 0.05.

First, we compared normal and MF CD34^+^ HSPCs in our in vitro extravasation model and demonstrated that MF HSPC migration is significantly more prominent (23,435 ± 11,499 migrated HD cells vs. 53,915 ± 21,093 migrated MF cells, *p* < 0.0001) in the presence of the same chemotactic stimulus (i.e., SDF‐1) (Figure 6a).

Next, we assessed the effect of CD44 inhibition on HSPC migration. While anti‐CD44 antibody causes only a small decrease in the number of migrated HD cells (Figure 6b), MF HSPCs are more sensitive to anti‐CD44 antibody as it significantly reduces MF cells migration in dose‐dependent manner (20 μg/mL 19.1% ± 12.10%, *p* = 0.0498; 40 μg/mL 27.6% ± 16.15%, *p* = 0.0081; Figure 6b). Moreover, the migration of MF HSPCs is significantly reduced as compared to HD cells treated with 40 μg/mL monoclonal anti‐CD44 antibody (respectively, 27.6% ± 16.15% vs. 7% ± 17.72%, *p* = 0.0220) (Figure 6b).

Collectively, our results suggest that CD44 is a key player in mediating HSPC extravasation and migration in MF.

### 3.7 HA Produced by MF Monocytes Drives HSPC Migration in vitro

To understand which of the two ligands of CD44, derived from monocytes, could promote HSPC migration in MF, we performed an ELISA assay on HD and MF monocyte conditioned medium after 96 h in vitro culture. We observed that MF monocytes secrete much more OPN and HA in vitro as compared with HD counterparts (Figure 6c,d), in agreement with increased OPN and HA plasma levels in MF patients.

To investigate the crosstalk between monocytes and HSPCs, we performed in vitro extravasation experiments with HD HSPCs using monocyte conditioned medium as a chemoattractant. We observed that MF monocytes are more effective in promoting cell migration as the number of migrated HSPCs per single monocyte conditioning the medium is significantly higher for MF samples compared with HD ones (Figure 6e). Moreover, CD44 inhibition reduces HSPC migration induced by MF monocyte conditioned medium (Figure 6f). We further investigated the involvement of CD44 ligands in this experimental setting and observed that only HA inhibition significantly reduces HSPC migration (Figure 6g). Supporting the prominent role of HA, we found that the number of migrated HSPCs positively correlates with HA concentration in monocyte conditioned medium (*p* = 0.0098) but not with OPN concentration (*p* = 0.3528) (Figure 6h,i).

Collectively, our results demonstrated that MF HSPCs display intrinsic properties of mobilisation when compared with HD ones and that CD44 increased expression could play a crucial role, as demonstrated by the reduction of migration induced by the anti‐CD44 antibody. MF monocytes secrete CD44 ligands, among which HA represents the most effective chemoattractant promoting HSPC migration.

## 4 Discussion

EMH is the generation of blood cells outside of the BM and is a hallmark of hematologic disorders, particularly MF, which among MPNs is characterised by the worst prognosis [34] and by the mobilisation of CD34^+^ cells from the BM [35, 36]. The presence of EMH results in the development of splenomegaly and impacts also disease progression, leukaemia free survival and overall survival because of the systemic spreading of the malignant clone [37]. However, as the mechanisms underlying EMH are poorly understood, we studied the pathogenesis of splenomegaly in MF, taking advantage of a MF mouse model and an in vitro extravasation assay.

Our leading hypothesis considers HSPCs as the main cell type involved in the onset of EMH, as they are the sole population responsible for producing blood cells; nevertheless, monocytes and macrophages may initiate EMH by producing pro‐inflammatory cytokines that reactivate splenic dormant niches and attract HSPCs from the BM. In TPO‐RA mice, that develop a condition recapitulating MF [19, 23], we observed the disruption of spleen architecture due to its colonisation by HSPCs that initiate EMH and lead to the accumulation of megakaryocytes and their immature progenitors. Moreover, F4/80^+^ macrophages were increased in the spleen of TPO‐RA treated mice, supporting the idea that they are involved in HSPC recruitment. This hypothesis is confirmed by the observation that in an inducible *Jak2* ^*V617F*^ transgenic mouse model, macrophage depletion through clodronate led to a 30% reduction in splenomegaly [28].

Although the JAK inhibitor Ruxolitinib can reduce spleen size in many MF patients, a significant percentage of patients do not respond to treatment [10, 14] and the systemic spreading of the neoplastic clone remains unaffected by the drug [38]. Therefore, a deeper understanding of the molecular and cellular processes involved in EMH and the identification of reinforcing strategies to inhibit EMH in MF patients has become an unmet need. In MF mice, Ruxolitinib treatment reduced the spleen index and, to a lesser extent, infiltrating macrophages, but was unable to eliminate HSPCs and megakaryocytes and to restore organ architecture. Accordingly, JAK inhibition of CD34^+^ cells derived from the spleen of MF patients did not affect disease propagating stem cells [13] suggesting its limited effect on EMH. To deepen the effect of Ruxolitinib on cell migration, we exploited an in vitro extravasation model based on a Transwell system coated with HUVEC cells. According to our results, Ruxolitinib does not affect human monocyte and HSPC migration from both HD and MF patients, therefore suggesting that the effects observed in TPO‐RA treated mice are due to its anti‐inflammatory activity [39]. Our previous works focused on the role of OPN in PMF as a driver of fibrosis development and progression [18, 19]. Nevertheless, OPN is a well‐known chemoattractant molecule for monocytes [30], and therefore we investigated the role of its receptors in cell migration, supported by the observation that Ruxolitinib causes a partial reduction of OPN plasma levels in MF mice. Flow cytometry evaluation of OPN receptor expression in monocytes and HSPCs from MF patients and HDs sustained the hypothesis that CD44 might play a pivotal role in regulating cell migration, as it is expressed by all monocytes and the percentage of MF CD44^+^ circulating HSPCs is significantly increased. Previous reports demonstrated the expression of CD44 and ⍺4 integrin in MF HSPCs isolated from the spleen [16]. Accordingly, CD44^+^ LSKs and macrophages were increased in MF mice spleens and CD44^+^ macrophages were only partially reduced upon Ruxolitinib treatment. Collectively, these results suggest that OPN may promote cell migration in the spleen of both MF mice and patients through its receptor CD44.

In vitro experiments confirmed the pivotal role of CD44 in regulating HD and MF monocyte migration. Indeed, anti‐CD44 antibodies were the most effective in reducing monocyte migration as compared to ⍺4β1 and αvβ3 inhibitors due to their ubiquitous expression in both HD and MF monocytes. Notably, the monoclonal anti‐CD44 antibody, which is an IM7 clone that can bind all CD44 isoforms recognising constant epitopes near the extracellular proximal domain [40], induced a dose‐dependent reduction in monocyte migration, up to its complete halt at the highest doses. In MF patients, endothelial cells take part in the microenvironment remodelling through the increased production of SDF and stem cell factor (SCF) which support haematopoietic progenitor cells' clonogenicity and long‐term culture [9]. Interestingly, we found that TNF‐⍺ activated endothelial cells lining the Transwell membrane enhance the effect of the anti‐CD44 antibody, thus suggesting that CD44 also mediates the interaction with the inflamed endothelium.

CD44 is a complex transmembrane adhesion glycoprotein that engages with diverse extracellular matrix components, including OPN and HA [41], and transduces intracellular signals crucial for cell proliferation, differentiation, and migration. CD44^−/−^ mice display normal development with no defects in haematopoiesis but altered trafficking of myeloid progenitors and leukocytes [42, 43]. CD44 is crucial for monocyte/macrophage migration, as demonstrated by a mouse model of experimentally induced autoimmune uveoretinitis (EAU) where the incubation of leukocytes with an anti‐CD44 monoclonal antibody before transplantation into recipient mice significantly suppressed monocyte rolling and reduced macrophage infiltration in the retinal parenchyma [40]. Moreover, it has been demonstrated that overexpression of CD44 significantly contributes to metastatic capacity in solid tumours [44]. In chronic lymphocytic leukaemia (CLL) CD44 is part of a surface molecular complex together with ⍺4β1, CD38, CXCR4, and MMP‐9 that is absent in normal B‐cells and coordinates adhesion and homing of CLL cells [45, 46]. Accordingly, CD44 and ⍺4β1 are both expressed by HD and MF monocytes; nevertheless, we demonstrated that CD44 inhibition is much more effective in reducing monocyte migration in vitro.

To unravel which CD44 ligand was primarily involved in promoting monocyte migration in the spleen of MF patients, we focused on OPN and HA, based on our previous finding concerning OPN in MF patients [18] and considering higher HA concentration in MF plasma samples compared with HDs. Our results demonstrated that, although both OPN and HA inhibition cause a slight reduction in monocyte migration in vitro, targeting CD44 directly achieves the strongest effect, and the concurrent inhibition of either of its two ligands only partially reinforces it.

It is well known that the frequency of circulating CD34^+^ HSPCs is increased in MF patients [47]. This is consistent with the hypothesis that EMH is caused by the activation of HSPCs migrated from the BM into the spleen. Here, we demonstrate that MF HSPCs migrate more efficiently in vitro and this correlates with CD44 increased expression. This is also in agreement with data demonstrating the reduced number of spleen‐derived myeloid colony‐forming units in CD44^−/−^ mice treated with granulocyte‐colony stimulating factor [42]. Our results highlight the pivotal role of CD44 in HSPC migration in the context of MF since anti‐CD44 monoclonal antibody caused a significant reduction in MF HSPCs migration but just a slight decline in the migration of HD cells, suggesting that neoplastic HSPCs are more sensitive to the anti‐CD44 treatment.

Monocytes and activated macrophages are thought to be responsible for the recruitment of HSPCs in the spleen through the production of chemoattractant molecules [26, 27, 28]. According to this, we demonstrated that MF monocytes are more effective in producing CD44 ligands, in particular OPN and HA, compared to HD cells. MF monocyte conditioned medium promoted in vitro HSPC migration more efficiently than HD monocyte conditioned medium and, in this setting, targeting CD44 is much more effective in reducing HSPC migration. Moreover, we demonstrated that HA inhibition decreases HSPC migration induced by MF monocyte conditioned medium, while targeting OPN, alone or in combination with HA, has no effect. Accordingly, the migration of HSPCs positively correlates with HA concentration in monocyte conditioned medium, but not with OPN concentration, suggesting that HA is the main CD44 ligand that regulates HSPC migration.

As a whole, our results demonstrate that CD44 and its ligands are involved in regulating migratory events responsible for the onset of EMH in MF patients, particularly by affecting monocyte and HSPC migration. Notably, the present work paves the way for future investigations to confirm that CD44 could be a new therapeutic target, which is already being tested in clinical trials for solid tumours (NCT01358903, NCT03078400, NCT02046928). In this light, the combo anti‐CD44‐Ruxolitinib could be considered a new therapeutic strategy to limit disease spreading in patients with MF.

## Author Contributions

**Margherita Mirabile:** conceptualization (equal), formal analysis (equal), investigation (equal), methodology (equal), writing – original draft (equal). **Camilla Tombari:** visualization (equal). **Anita Neroni:** visualization (equal). **Lara Tavernari:** methodology (equal). **Ruggiero Norfo:** funding acquisition (equal), investigation (equal). **Elisa Bianchi:** funding acquisition (equal), methodology (equal). **Monica Maccaferri:** resources (equal). **Barbara Mora:** resources (equal). **Sandra Parenti:** investigation (equal). **Chiara Carretta:** formal analysis (equal). **Matteo Bertesi:** formal analysis (equal). **Marica Malerba:** investigation (equal). **Elisa Papa:** investigation (equal). **Luca Fabbiani:** investigation (equal). **Niccolò Bartalucci:** investigation (equal). **Paola Guglielmelli:** supervision (equal). **Leonardo Potenza:** resources (equal). **Lorena Losi:** supervision (equal). **Francesco Passamonti:** resources (equal). **Enrico Tagliafico:** supervision (equal), writing – original draft (equal). **Mario Luppi:** resources (equal), supervision (equal). **Sebastiano Rontauroli:** formal analysis (equal), visualization (equal), writing – original draft (equal). **Alessandro Maria Vannucchi:** supervision (equal). **Rossella Manfredini:** conceptualization (equal), funding acquisition (equal), supervision (equal), writing – original draft (equal).

## Ethics Statement

The study was performed in accordance with the Declaration of Helsinki and was approved by the local ethical committee (Comitato Etico dell'Area Vasta Emilia Nord, AVEN). Animal studies were reviewed and approved by the Italian Ministry of Health.

## Consent

All subjects provided informed written consent.

## Conflicts of Interest

The authors declare no conflicts of interest.

## Supporting information


**Figure S1.** TPO‐RA treatment remodels spleen architecture. (A) Stack images of haematoxylin–eosin stained spleen sections from controls (NT), TPO‐RA treated mice (TPO‐RA) and MF mice receiving Ruxolitinib (TPO‐RA + Ruxo). Whole spleen section stack images, one representative mouse from each group, is included as well as half‐spleen images for the remaining animals. (B) Representative stack images of haematoxylin–eosin stained spleen sections from one representative mice for each group are compared with F4/80 immunohistochemistry from the same animals. F4/80 is a surface marker for macrophages that reside within red pulp regions of the spleen. Immunohistochemistry demonstrates macrophages are excluded from white pulp regions in control mice, TPO‐RA treated animals and MF mice who received Ruxolitinib. 200 μm scale bar is shown.
**Figure S2.** Gating strategy for the immunophenotypic characterisation of LSK, MKP and macrophages in mice spleens. Panels (A–C) report the gating strategy for LSK, MKP and macrophages respectively. Starting from the selected all events gate, doublets and dead cells were excluded. LSK cells were identified within lineage negative population as Sca1 positive and c‐Kit positive events (A). Among lineage negative cells, MKP were identified as Sca1 negative c‐Kit positive CD150 positive CD41 positive cells (B). Within live cells, macrophages were identified as CD11b positive F4/80 positive cells (C). In both LSK and macrophages we evaluated the expression of CD49d (α4 integrin), CD29 (β1 integrin), CD51 (αv integrin), CD61 (β3 integrin) and CD44 as reported. In each graph, the frequency of the selected population is reported. A representative sample is shown.
**Figure S3.** Results of preliminary experiment conducted to define optimal conditions for in vitro migration assay. (A) Bar graph displays results of a preliminary experiment conducted to define HUVEC number, TNF‐α concentration and CD14^+^ cell number. Each condition is reported within the table below the graph. The TNF‐α concentration is also reported as colour code (green for 50 μg/mL, yellow for 100 μg/mL). The sample that did not include HUVEC is indicated in grey. *Y* axis reports the cell count obtained using counting beads for flow cytometric analysis. (B) Graph represents results of a preliminary test conducted to setup the optimal number of CD34^+^ cells, culture medium and the presence of SDF‐1 in migration experiments. The medium and the presence of SDF‐1 are reported with labels on *X* axis. The number of CD34^+^ cells is indicated with pink bar colour for 100,000 cells while blue bar colour stands for 200,000 cells loaded on Transwell system. All samples included HUVEC coating. RPMI indicates RPMI +0.25% BSA while IMDM stands for IMDM +10% FBS + L‐glu.
**Figure S4.** Gating strategy for the immunophenotypic characterisation of HD PBMCs. In the represented flow cytometry plots starting from the selected all events, doublets and dead cells were excluded. Among the remaining events we identified CD14^+^ and CD34^+^ cells. In both CD14^+^ and CD34^+^ cells we evaluated the expression of αvβ3, α4, β1, CD44 and CD44v6. In each graph, the frequency of the selected population is reported. A representative sample is shown.
**Figure S5.** Gating strategy for the immunophenotypic characterisation of MF PBMCs. In the represented flow cytometry plots starting from the selected all events, doublets and dead cells were excluded. Among the remaining events we identified CD14^+^ and CD34^+^ cells. In both CD14^+^ and CD34^+^ cells we evaluated the expression of αvβ3, α4, β1, CD44 and CD44v6. In each graph, the frequency of the selected population is reported. A representative sample is shown.
**Figure S6.** XTT assay results. The red dashed line represents 70% viability. Panels (A–C) show XTT assay results evaluating cytotoxicity of Ruxolitinib and the relative control DMSO in HD CD14^+^ cells and HD CD34^+^ cells. Panels (D–G) display results of control IgG cytotoxicity evaluation in HD CD14^+^ cells as compared to not treated cells. Panels (H–K) show XTT assay results evaluating antibodies recognising OPN receptors cytotoxicity in HD CD14^+^ cells as compared to control IgG samples at the same concentration. Panel (L, M) display XTT assay results for the monoclonal anti‐CD44 antibody and corresponding control IgG in HD CD34^+^ cells. Comparisons between the treated samples and the control were performed by means of paired *T*‐test. *p*‐values are reported only if < 0.05.
**Figure S7.** Migration results for the monoclonal anti‐⍺4β1 antibodies with and without HUVEC on CD14^+^ cells. Bar plot shows the effect on CD14^+^ cells migration due to the monoclonal anti‐α4β1. Transwell samples with HUVEC are represented with purple bars while Transwell samples without HUVEC are illustrated with pink bars. The red dashed line represents 100% migration; the percentage of migration is normalised considering control IgG samples, not shown in this figure. Comparisons were performed by means of paired *T*‐test.
**Table S1.** The list of MF patients included in the study with the corresponding clinical features. The figures where the patient sample was used is indicated.

## Data Availability

The data that support the findings of this study, protocols, and methods are available in the main text and Supporting Information.
