# Supplementary material for: CD44 Participates to Extramedullary Haematopoiesis Onset by Mediating the Interplay Between Monocytes and Haematopoietic Stem Cells in Myelofibrosis
Source: J Cell Mol Med. 2025 Jul 21;29(14):e70720. doi: 10.1111/jcmm.70720 (PMC12279041; doi:10.1111/jcmm.70720)
Supplement: Supplementary file 1 — Figure S1. TPO‐RA treatment remodels spleen architecture. (A) Stack images of haematoxylin–eosin stained spleen sections from controls (NT), TPO‐RA treated mice (TPO‐RA) and MF mice receiving Ruxolitinib (TPO‐RA + Ruxo). Whole spleen section stack images, one representative mouse from each group, is included as well as half‐spleen images for the remaining animals. (B) Representative stack images of haematoxylin–eosin stained spleen sections from one representative mice for each group are compared with F4/80 immunohistochemistry from the same animals. F4/80 is a surface marker for macrophages that reside within red pulp regions of the spleen. Immunohistochemistry demonstrates macrophages are excluded from white pulp regions in control mice, TPO‐RA treated animals and MF mice who received Ruxolitinib. 200 μm scale bar is shown. Figure S2. Gating strategy for the immunophenotypic characterisation of LSK, MKP and macrophages in mice spleens. Panels (A–C) report the gating strategy for LSK, MKP and macrophages respectively. Starting from the selected all events gate, doublets and dead cells were excluded. LSK cells were identified within lineage negative population as Sca1 positive and c‐Kit positive events (A). Among lineage negative cells, MKP were identified as Sca1 negative c‐Kit positive CD150 positive CD41 positive cells (B). Within live cells, macrophages were identified as CD11b positive F4/80 positive cells (C). In both LSK and macrophages we evaluated the expression of CD49d (α4 integrin), CD29 (β1 integrin), CD51 (αv integrin), CD61 (β3 integrin) and CD44 as reported. In each graph, the frequency of the selected population is reported. A representative sample is shown. Figure S3. Results of preliminary experiment conducted to define optimal conditions for in vitro migration assay. (A) Bar graph displays results of a preliminary experiment conducted to define HUVEC number, TNF‐α concentration and CD14+ cell number. Each condition is reported within the table be [file JCMM-29-e70720-s001.docx]

**SUPPLEMENTARY MATERIALS**

**SUPPLEMENTARY METHODS**

**Human CD34^+^ and CD14^+^ cell purification**

Human peripheral blood mononuclear cells (PBMC) were collected using a density gradient. Briefly, blood sample was diluted 1:5 with Phosphate-Buffered Saline (PBS) with 2 mM EDTA, stratified over Ficoll–Paque gradient (Lympholyte^®^; Cederlane Labs) and centrifuged at 800xg for 20 minutes at room temperature (RT) in a swinging-bucket rotor without brake. Following centrifugation, PBMCs were recovered and washed thrice with PBS. CD34 or CD14 positive cells were isolated from PBMCs through immunomagnetic positive selection using the corresponding MicroBead Kits UltraPure (MILTENYI BIOTEC, Bergish Gladbach, Germany. Freshly isolated CD14^+^ and CD34^+^ cells from healthy donors (HD) as well as CD14^+^ cells from myelofibrosis (MF) patients were used for experiments; MF CD34^+^ cells were frozen and cryopreserved in Fetal Bovine Serum (FBS, Sigma-Aldrich, Merck, Darmstadt, Germany) supplemented with 10% DMSO (ThermoFisher, Waltham, Massachussets, USA).

**Cell culture conditions**

Human Umbilical Endothelial Cells (HUVECs) were purchased from ThermoFisher (catalog number #C-003-5C, Waltham, Massachussets, USA). After thawing, HUVECs were seeded with Medium 200 supplemented with Low Serum Growth Supplement (ThermoFisher, Waltham, Massachussets, USA). For HUVECs detachment, Trypsin/EDTA (3 minutes at RT) and then Trypsin Neutralizer from ThermoFisher (Waltham Massachussets, USA) were used.

CD14^+^ cells were seeded in Iscove’s Modified Dulbecco Medium (IMDM, Euroclone, Milano, Italy) supplemented with 10% FBS HyClone (GE Healthcare Life Sciences, Utah, USA), 100 U/ml Penicillin, 100 ug/ml Streptomycin (Euroclone, Milano, Italy), 292 ug/ml L-Glutamine (Euroclone, Milano, Italy) and 10 ng/ml human granulocyte-macrophage colony-stimulating factor (GM-CSF, Miltenyi Biotech, Bergisch Gladbach, Germany).

Cryopreserved MF CD34^+^ cells were thawed with heated IMDM supplemented with 10% FBS, P/S and L-Glutamine. MF CD34^+^ cells, as well as freshly isolated HD CD34+ cells, were seeded at 1x10^6^ cells/ml in IMDM supplemented with 10% FBS, P/S and L-Glutamine, and the following cytokines were added to preserve stem cell properties: human stem cell factor (SCF) 50 ng/ml, human Flt3-Ligand (FLT3L) 50 ng/ml, human thrombopoietin (TPO) 20 ng/ml, human interleukin-3 (IL3) 10 ng/ml and human interleukin-6 (IL6) 10 ng/ml (all from Miltenyi Biotec, Bergisch Gladbach, Germany).

**Immunophenotyping**

Immunophenotyping was performed using the following antibodies: PE conjugated anti-humanCD14 antibody 1:50 (cat. #130-113-147, Miltenyi Biotec, Bergisch Gladbach, Germany); BV421 conjugated anti-humanCD34 antibody 1:80 (cat. #343610, BD Biosciences, New York, USA); APC conjugated anti-humanαvβ3 antibody 1:80 (cat. 304416, BD Biosciences, New York, USA); APCCy7 conjugated anti-humanα4 antibody 1:80 (cat. #304327, BD Biosciences, New York, USA); PerCPCy5.5 conjugated anti-humanβ1 antibody 1:80 (cat. #303023, BD Biosciences, New York, USA); FITC conjugated anti-humanCD44 1:160 (cat. #560977, BD Biosciences, New York, USA); PE-Vio770 conjugated anti-humanCD44v6 1:100 (cat. #130-11-426, Miltenyi Biotec, Bergisch Gladbach, Germany); BV605 conjugated anti-mouseSca1 antibody 1:400 (cat. #108134, BioLegend, San Diego, CA, US); BV711 conjugated anti-mouseCD44 antibody 1:640 (cat. #103057, BioLegend, San Diego, CA, US); FITC conjugated anti-mouseCD29 antibody 1:160 (cat. #102205, BioLegend, San Diego, CA, US); PE conjugated anti-mouseCD51 antibody 1:640 (cat. #104105, BioLegend, San Diego, CA, US); PE/Dazzle594 conjugated anti-mouseCD49d antibody 1:160 (cat. #103625, BioLegend, San Diego, CA, US); PE-Cy5 conjugated anti-mouseCD4 antibody 1:800 (cat. #100514, BioLegend, San Diego, CA, US); PE-Cy5 conjugated anti-mouseCD5 antibody 1:600 (cat. #100610, BioLegend, San Diego, CA, US); PE-Cy5 conjugated anti-mouseCD8 antibody 1:1600 (cat. #100710, BioLegend, San Diego, CA, US); PE-Cy5 conjugated anti-mouseCD11b antibody 1:1600 (cat. #101210, BioLegend, San Diego, CA, US); PE-Cy5 conjugated anti-mouseB220 antibody 1:800 (cat. #103209, BioLegend, San Diego, CA, US); PE-Cy5 conjugated anti-mouseTer119 antibody 1:800 (cat. #116210, BioLegend, San Diego, CA, US); PE-Cy5 conjugated anti-mouseLy-6G/Ly-6C antibody 1:400 (cat. #108409, BioLegend, San Diego, CA, US); APC conjugated anti-mouseCD61 antibody 1:640 ; APC-ef780 conjugated anti-mouseCD117 antibody 1:1600 (cat. #47-1171-82, eBioscience, ThermoFisher, Waltham, Massachussets, USA); BV786 conjugated anti-mouseF4/80 antibody 1:150 (cat. #123141, BioLegend, San Diego, CA, US); PE-Cy7 conjugated anti-mouseCD11b antibody 1:1000 (cat. #101216, BioLegend, San Diego, CA, US); BV785 conjugated anti-mouseCD150 antibody 1:1600 (cat. #115937, BioLegend, San Diego, CA, US); PE-Cy7 conjugated anti-mouseCD41 antibody 1:1600 (cat. #25-0411-80, eBioscience, ThermoFisher, Waltham, Massachussets, USA).

Since no single antibody against α4β1 integrin complex was available, after the staining with individual antibodies against α4 and β1 monomers we considered double positive (α4^+^β1^+^) cells as positive to α4β1 (Supplemental Figures 1 and 2). To exclude dead cells, we stained samples with LIVE/DEAD™ Fixable Aqua Dead Cell Stain Kit, for 405 nm excitation, (ThermoFisher, Waltham, Massachussets, USA) or DAPI (Miltenyi Biotec, Bergisch Gladbach, Germany) (Supplemental Figures 2, 4 and 5).

Briefly, after washing with PBS and centrifugation at 300g for 10 minutes, we added LIVE/DEAD™ diluted 1:100 in PBS and incubated at room temperature in the dark for 10 minutes. After a second wash with PBS and a centrifugation at 300g for 10 minutes, we added FcR blocking 1:5 in PBS supplemented with 2 mM EDTA and 2% FBS (subsequently reported in this paragraph simply as staining buffer) and incubated cells at 4°C in the dark for 10 minutes. After the addition of antibodies, samples were incubated at 4°C in the dark for 20 minutes. After a final wash with staining buffer and centrifugation at 300g for 10 minutes, the samples were resuspended in staining buffer and evaluated by flow cytometry. Supplementary Figure 2 displays the adopted gating strategy for the characterization of cells from MF mice spleens. Supplementary Figure 4 reports the adopted gating strategy for the characterization of CD14^+^ and CD34^+^ cells in HD samples. Supplementary Figure 5 shows the adopted gating strategy for MF sample.

**Viability assay**

We carried out viability tests with TACS^®^ XTT Cell Proliferation Assay, cat. #4891-025-K, R&D systems (Minneapolis, Minnesota, USA). After CD14^+^ and CD34^+^ cell purification, CD14^+^ cells were seeded in 96-well culture plates at 260,000 cells/well density and CD34^+^ cells were seeded in 96-well culture plates at 100,000 cells/well density. After an overnight cell culture with the tested antibody or drug, the XTT assay was performed following manufacturer instructions.

**Enzyme-linked immunosorbent assay (ELISA)**

The levels of OPN in plasma samples from TPO-RA-treated mice were analyzed by means of ELISA using mouse/rat OPN Quantikine kit (cat. #MOST00, R&D Systems, Minneapolis, MN) according to the manufacturer’s instructions.

The levels of OPN and HA in CD14^+^ supernatants and the levels of HA in plasma samples from HDs and MF patients were detected with ELISA assay using human OPN Quantikine kit (cat. #DOST00) and Hyaluronan Quantikine kit (cat. #DHYAL0, both from R&D Systems, Minneapolis, MN) respectively, according to the manufacturer’s instructions. CD14+ monocytes were cultured as previously described [1] and detailed in Supplementary materials and conditioned culture medium was collected after 96 hours.

**SUPPLEMENTARY RESULTS**

***In vitro* system setup for the evaluation of CD14^+^ and CD34^+^ cell migration**

To setup optimal conditions for evaluating *in vitro* extravasation, we performed preliminary experiments to define the number of HUVEC cells to be used to coat the Transwell membrane, the concentration of TNF-α to be used to activate HUVEC cells, the number of CD14^+^ and CD34^+^ cells to be loaded.

First, we tested the number of HUVEC cells to be added on Transwell membrane in two conditions: 70’000 or 80’000 cells/Transwell. To obtain this coating, each Transwell was placed upside down in a culture plate with HUVEC cell suspension on top and incubated overnight. Next, we looked for the more effective TNF-α concentration to activate the HUVEC layer; we compared the effect of 50 ug/ml and 100 μg/ml TNF-α treatment. Finally, we set up the number of CD14^+^ cells to be loaded in Transwell upper chamber by comparing three conditions: 500’000, 650’000 or 800’000 CD14^+^ cells. Cells were then seeded on the Transwell insert coated with HUVEC cells and incubated overnight. FBS supplemented to the medium in the lower chamber served as chemoattractant for CD14^+^ cells.

Our results demonstrated that the number of migrated CD14^+^ cells raises as the number of cells loaded on the Transwell insert increases (Supplementary Figure 3 A): the highest recovery of CD14^+^ cells is achieved after the loading of 800’000 cells. Moreover, TNF-α 100 μg/ml induced a slight increase in migrated cells as compared to the corresponding samples treated with TNF-α 50 μg/ml. The same situation also occurs for the number of HUVEC cells: the presence of 80’000 HUVEC cells allow a more effective CD14^+^ cell migration as compared to 70’000 HUVEC cells per Transwell.

The comparison between samples loaded with 800’000 CD14^+^ cells with and without HUVEC cells clearly suggests that activated HUVEC cells, treated with TNF-α, promote monocyte migration, indeed the presence of cell coating allowed us to double the number of migrated monocytes (147’027 vs 74’531 counted cells, respectively).

Based on these results, subsequent experiments were conducted by loading 800’000 CD14^+^ cells; the Transwell insert was covered with 80’000 HUVEC cells activated with 100 μg/ml TNF-α.

For *in vitro* migration experiments using CD34^+^ cell, the same number of HUVEC cells and TNF-α concentration were used. We then tested the number of CD34^+^ cells to be loaded on Transwell system (100’000 or 200’000 CD34^+^ cells), the culture medium and the presence of the chemoattractant SDF-1. The two media tested were RPMI supplemented with BSA 0.25% and IMDM supplemented with FBS 10% and L-Gln. SDF-1 was used as chemoattractant for CD34^+^ cells.

Our results shown in Supplementary Figure 3 B demonstrated that the best condition is to seed 200’000 CD34+ cells/well in IMDM with SDF-1 as chemoattractant since it allowed us to obtain the highest number of migrated CD34+ cells. However, we decided to perform our experiments by plating 100’000 CD34+ cells/well in IMDM medium supplemented with SDF-1 because the number of migrated cells is sufficient to observe any difference and the amount of seeded CD34^+^ cells is compatible with the reduced cell availability compared to CD14^+^ monocytes.

The comparison between Transwell samples loaded with 100’000 CD34^+^ cells with and without HUVEC coating clearly suggests that HUVEC cells promote CD34^+^ cells migration too. Indeed, the presence of cell coating allowed us to double the number of migrated CD34^+^ cells (13’002 counted cells vs 5’660 counted cells, respectively).

**Viability assay after treatment with Ruxolitinib and antibodies against OPN receptors**

To test any possible effect on cell viability induced by Ruxolitinib or the OPN receptor neutralizing antibodies tested in *in vitro* migration assays we performed XTT assays on HD CD14^+^ and CD34^+^ cells. As a general rule, a treatment is considered cytotoxic when at least a 30% reduction in viability is induced [2] [3].

Because Ruxolitinib was resuspended in DMSO for *in vitro* testing, this solvent was tested for its cytotoxicity at the concentrations reached in the different Ruxolitinib doses (Supplementary Figure 4 A). Considering the effect of DMSO 0.0024% and DMSO 0.006% negligible, Ruxolitinib 0.2 uM and Ruxolitinib 0.5 uM were compared with not treated cells as controls. XTT assay was performed for Ruxolitinib in both HD CD14^+^ and HD CD34^+^ cells. The DMSO, at tested concentrations, was not toxic as compared to not treated cells (Supplementary Figure 4 A). The three highest doses of Ruxolitinib were toxic for CD14^+^ cells and exceeded the 70% vitality threshold (Supplementary Figure 4 B). Conversely, Ruxolitinib did not impact CD34^+^ cell viability at tested concentrations (1 uM and 5 uM) since normalized absorbances were almost identical to that observed in control sample (Supplementary Figure 4 C).

Antibodies recognizing OPN receptors were then tested and correlated with their control IgG at the respective concentrations. All IgG isotype controls were not toxic for CD14^+^ cells (Supplementary Figure 4 D, E, F and G). Control IgG for the monoclonal anti-CD44 antibody significantly increased CD14^+^ cell viability, which was about 50% higher as compared to not treated sample (Supplementary Figure 4 E). More interestingly, our results demonstrated that anti-OPN receptor antibodies are not toxic for CD14^+^ cells because cell viability is always over 70%.

XTT viability assays were conducted also on HD CD34^+^ cells. Control IgG induced a statistically significant increase in CD34^+^ cells viability as compared to not treated cells (Supplementary Figure 5, L). According to our results, the monoclonal anti-CD44 antibody does not affect CD34^+^ cell viability (Figure 5, panel J).

References

1. Bianchi, E.; Rontauroli, S.; Tavernari, L.; Mirabile, M.; Pedrazzi, F.; Genovese, E.; Sartini, S.; Dall’Ora, M.; Grisendi, G.; Fabbiani, L.; et al. Inhibition of ERK1/2 Signaling Prevents Bone Marrow Fibrosis by Reducing Osteopontin Plasma Levels in a Myelofibrosis Mouse Model. *Leukemia* **2023**, *37*, 1068–1079, doi:10.1038/s41375-023-01867-3.

2. GP ISO10993-In Vitro Cytotoxicity Test.Pdf.

3. Cannella, V.; Altomare, R.; Chiaramonte, G.; Di Bella, S.; Mira, F.; Russotto, L.; Pisano, P.; Guercio, A. Cytotoxicity Evaluation of Endodontic Pins on L929 Cell Line. *Biomed Res Int* **2019**, *2019*, 3469525, doi:10.1155/2019/3469525.

**SUPPLEMENTARY FIGURES AND TABLES**

**Supplementary Figure 1**

**
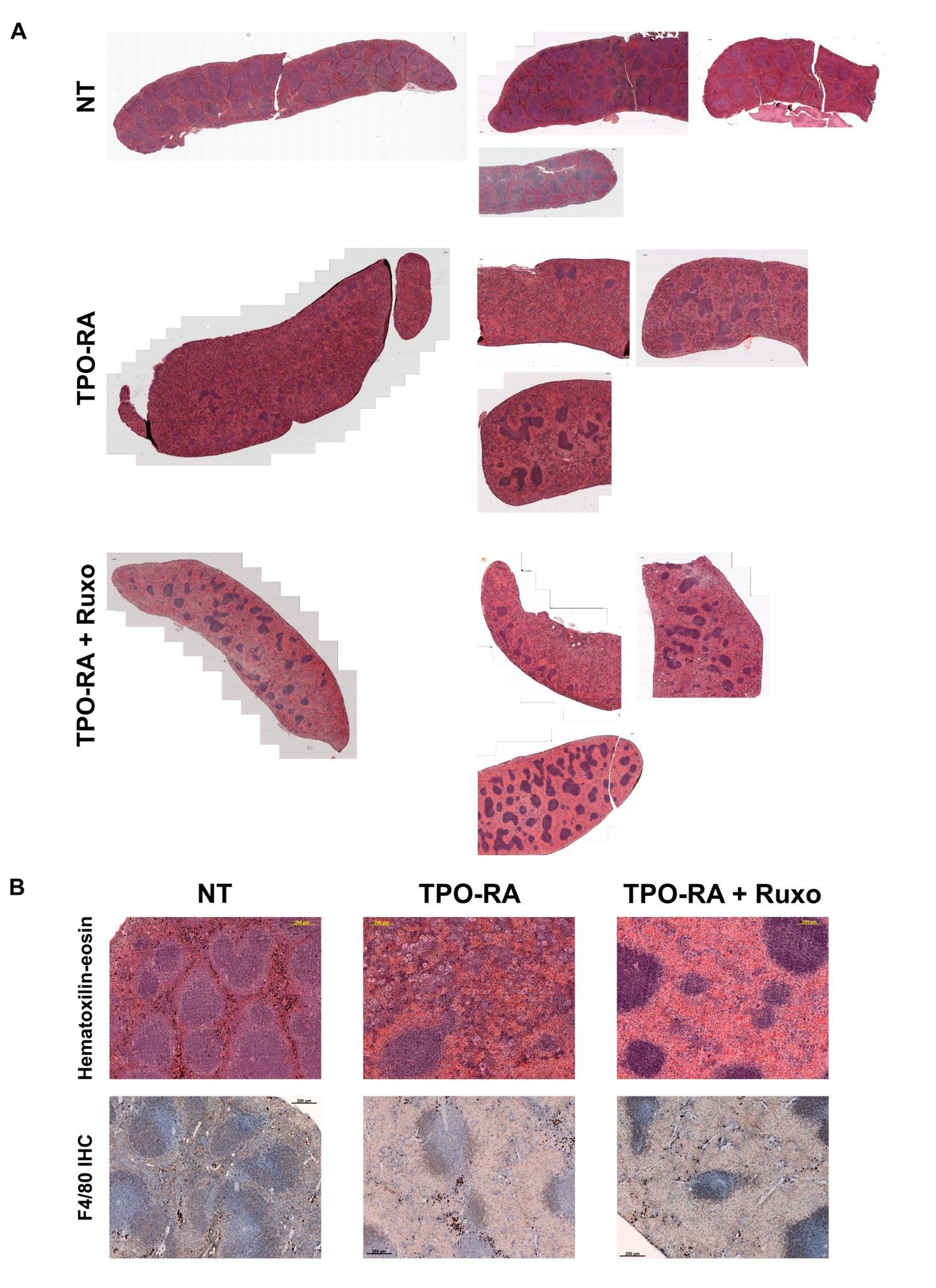
**

**Supplementary Figure 1: TPO-RA treatment remodels spleen architecture. A.** Stack images of hematoxylin-eosin stained spleen sections from controls (NT), TPO-RA treated mice (TPO-RA) and MF mice receiving Ruxolitinib (TPO-RA + Ruxo). Whole spleen section stack images, one representative mouse from each group, is included as well as half-spleen images for the remaining animals. **B.** Representative stack images of hematoxylin-eosin stained spleen sections from one representative mice for each group are compared with F4/80 immunohistochemistry from the same animals. F4/80 is a surface marker for macrophages that reside within red pulp regions of the spleen. Immunohistochemistry demonstrates macrophages are excluded from white pulp regions in control mice, TPO-RA treated animals and MF mice who received Ruxolitinib. 200 um scale bar is shown.

**Supplementary Figure 2**

**
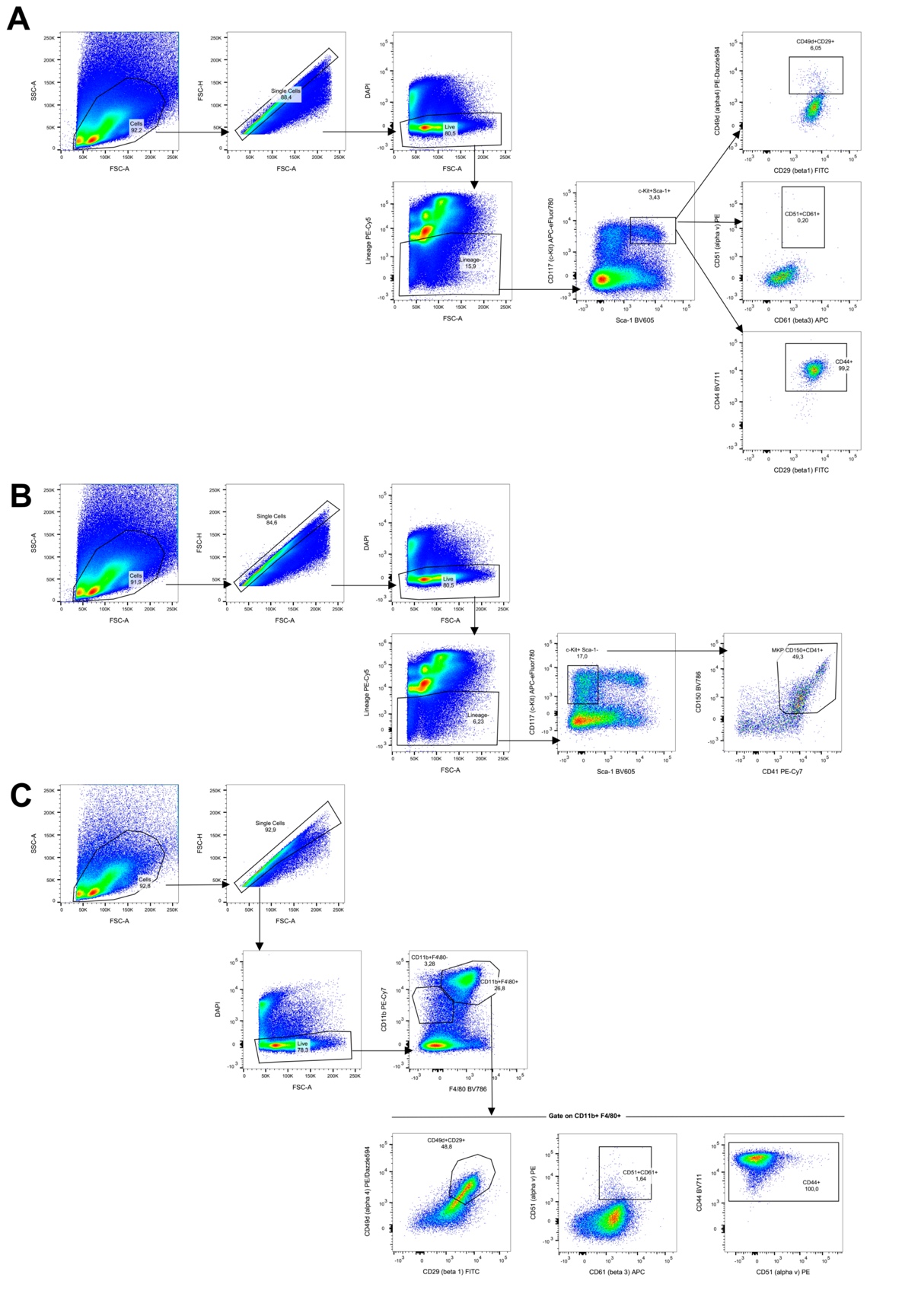
**

**Supplementary Figure 2:** **Gating strategy for the immunophenotypic characterization of LSK, MKP and macrophages in mice spleens.** Panels A, B and C report the gating strategy for LSK, MKP and macrophages respectively. Starting from the selected all events gate, doublets and dead cells were excluded. LSK cells were identified within lineage negative population as Sca1 positive and c-Kit positive events (A). Among lineage negative cells, MKP were identified as Sca1 negative c-Kit positive CD150 positive CD41 positive cells (B). Within live cells, macrophages were identified as CD11b positive F4/80 positive cells (C). In both LSK and macrophages we evaluated the expression of CD49d (α4 integrin), CD29 (β1 integrin), CD51 (αv integrin), CD61(β3 integrin), and CD44 as reported. In each graph, the frequency of the selected population is reported. A representative sample is shown.

**Supplementary Figure 3**

**
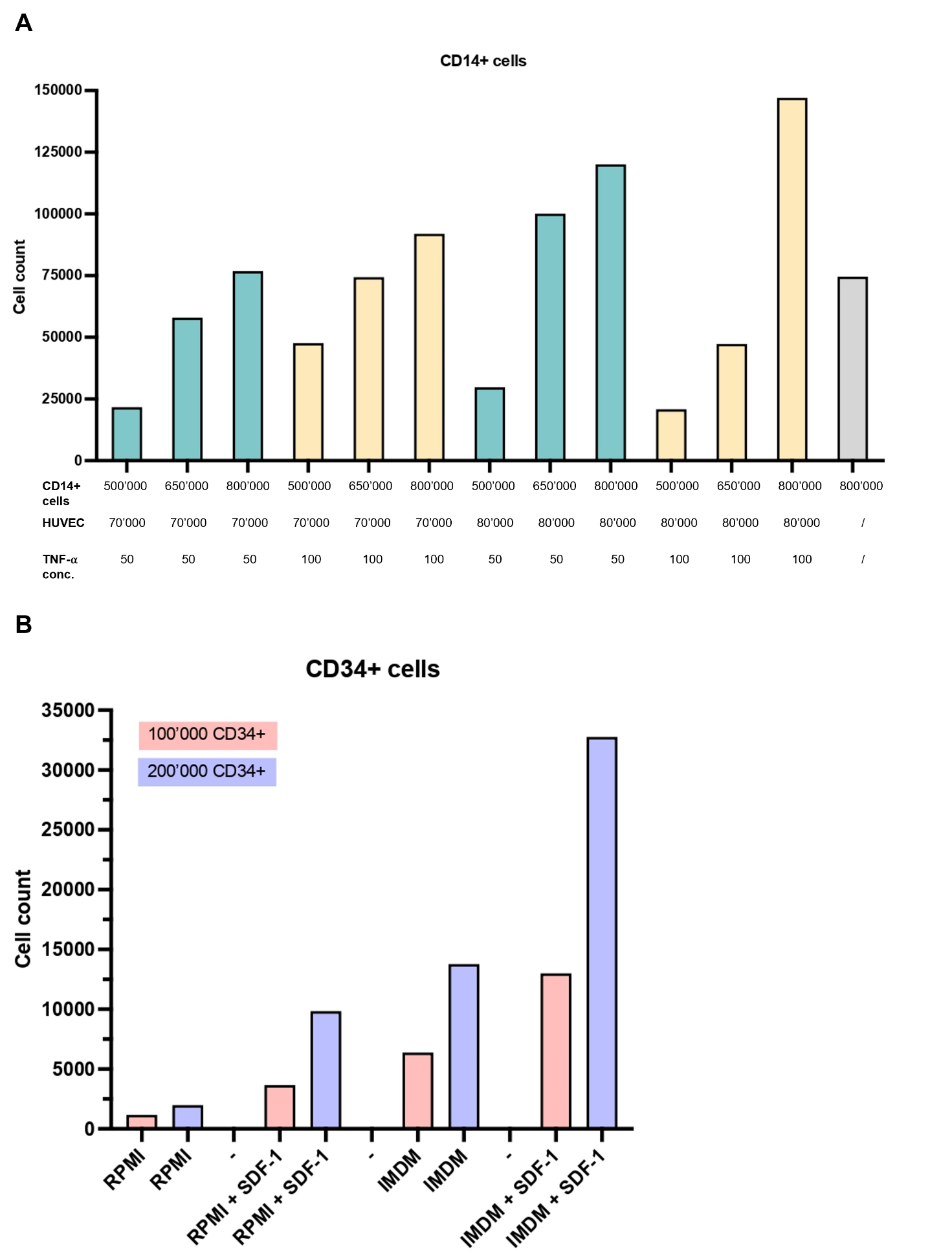
**

**Supplementary Figure 3: Results of preliminary experiment conducted to define optimal conditions for *in vitro* migration assay. A** Bar graph displays results of a preliminary experiment conducted to define HUVEC number, TNF-α concentration and CD14+ cell number. Each condition is reported within the table below the graph. The TNF-α concentration is also reported as color code (green for 50 μg/ml, yellow for 100 μg/ml). The sample that did not include HUVEC is indicated in grey. Y axis reports the cell count obtained using counting beads for flow cytometric analysis. **B** Graph represents results of a preliminary test conducted to setup the optimal number of CD34^+^ cells, culture medium and the presence of SDF-1 in migration experiments. The medium and the presence of SDF-1 are reported with labels on X axis. The number of CD34^+^ cells is indicated with pink bar color for 100’000 cells while blue bar color stands for 200’000 cells loaded on Transwell system. All samples included HUVEC coating. Abbreviations: RPMI indicates RPMI + 0.25% BSA while IMDM stands for IMDM + 10% FBS + L-glu.

**Supplementary Figure 4**


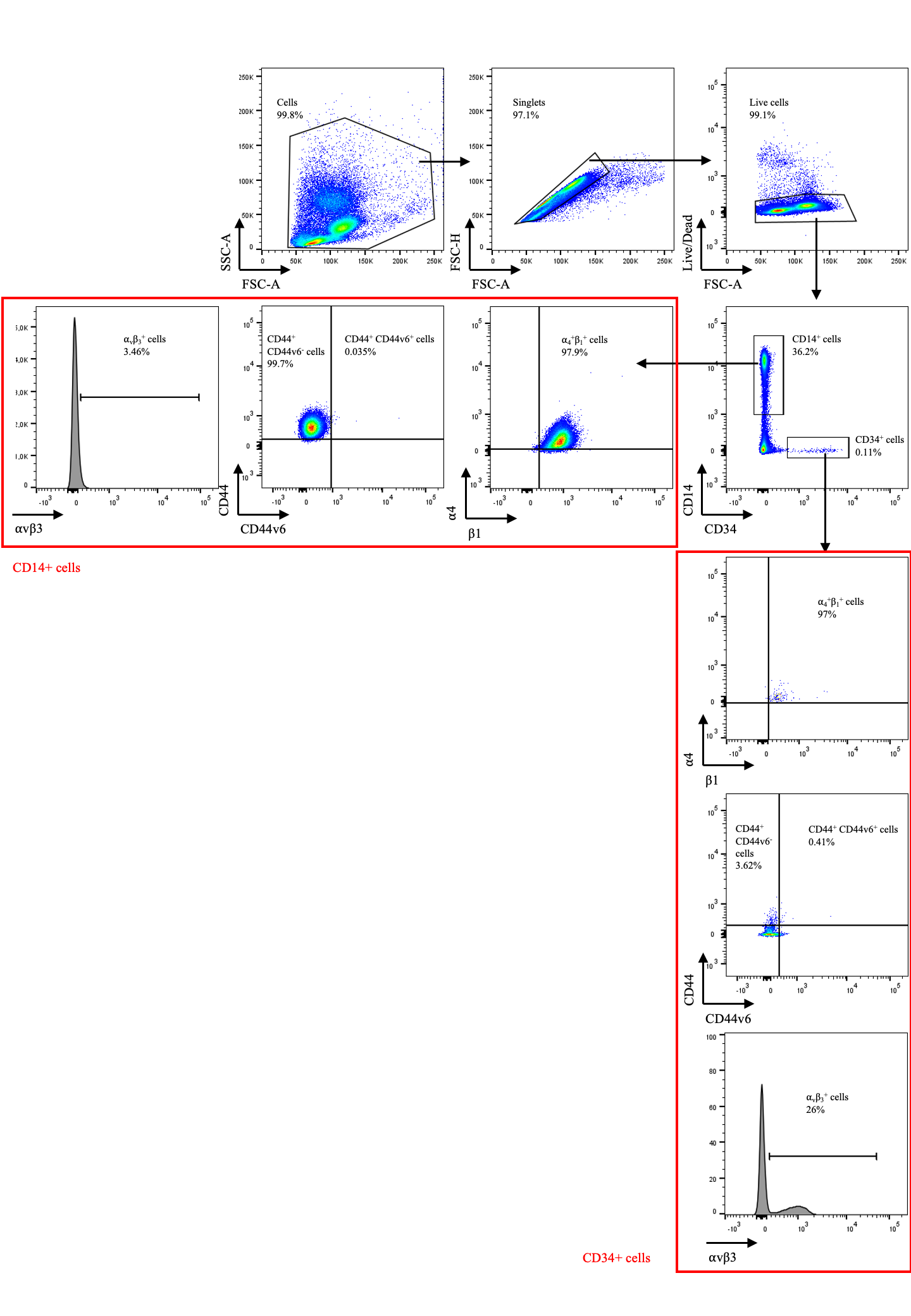


**Supplementary Figure 4:** **Gating strategy for the immunophenotypic characterization of HD PBMCs.** In the represented flow cytometry plots starting from the selected all events, doublets and dead cells were excluded. Among the remaining events we identified CD14+ and CD34+ cells. In both CD14+ and CD34+ cells we evaluated the expression of αvβ3, α4, β1, CD44 and CD44v6. In each graph, the frequency of the selected population is reported. A representative sample is shown.

**Supplementary Figure 5**


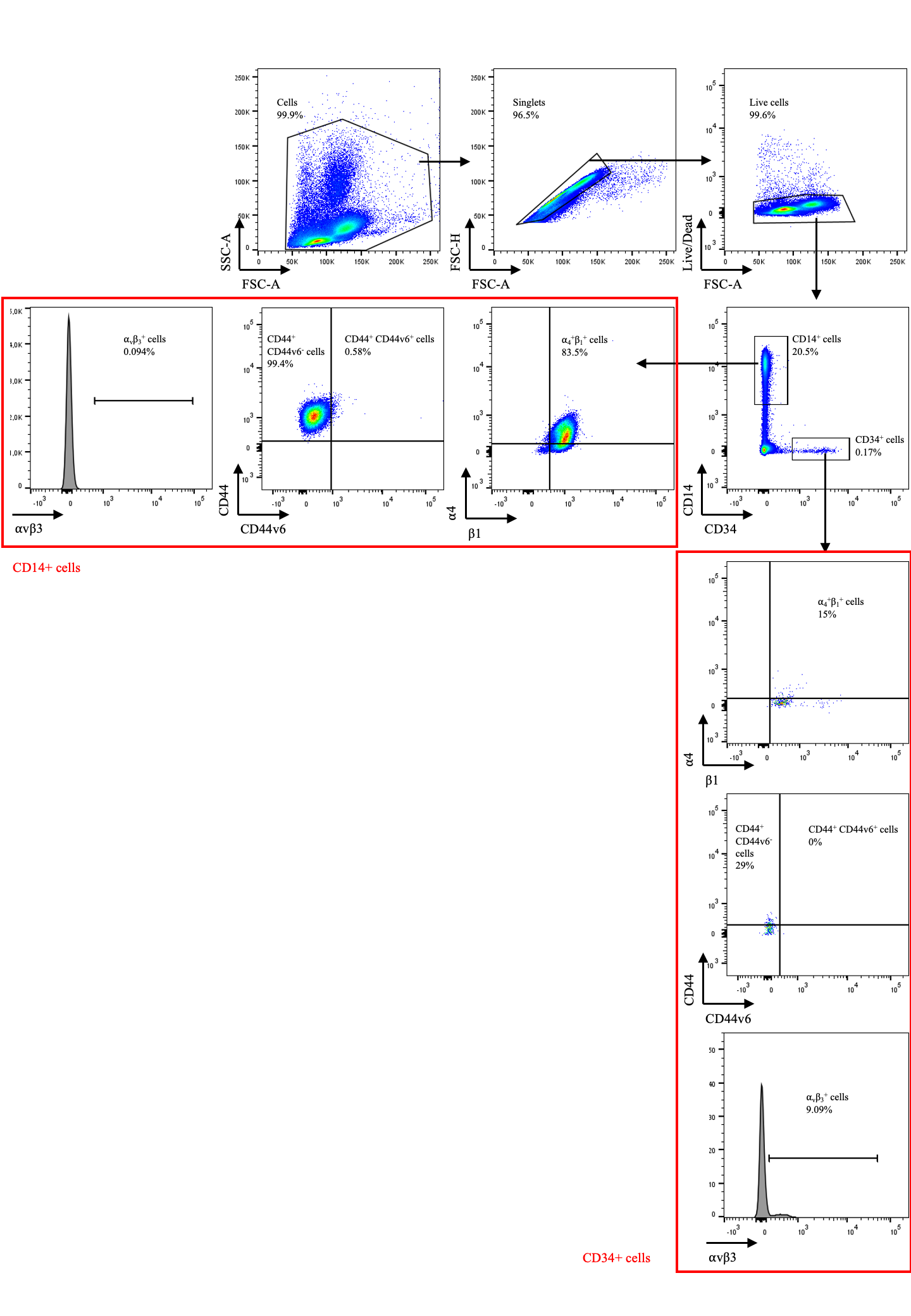


**Supplementary Figure 5:** **Gating strategy for the immunophenotypic characterization of MF PBMCs.** In the represented flow cytometry plots starting from the selected all events, doublets and dead cells were excluded. Among the remaining events we identified CD14^+^ and CD34^+^ cells. In both CD14+ and CD34+ cells we evaluated the expression of αvβ3, α4, β1, CD44 and CD44v6. In each graph, the frequency of the selected population is reported. A representative sample is shown.

**Supplementary Figure 6**

**
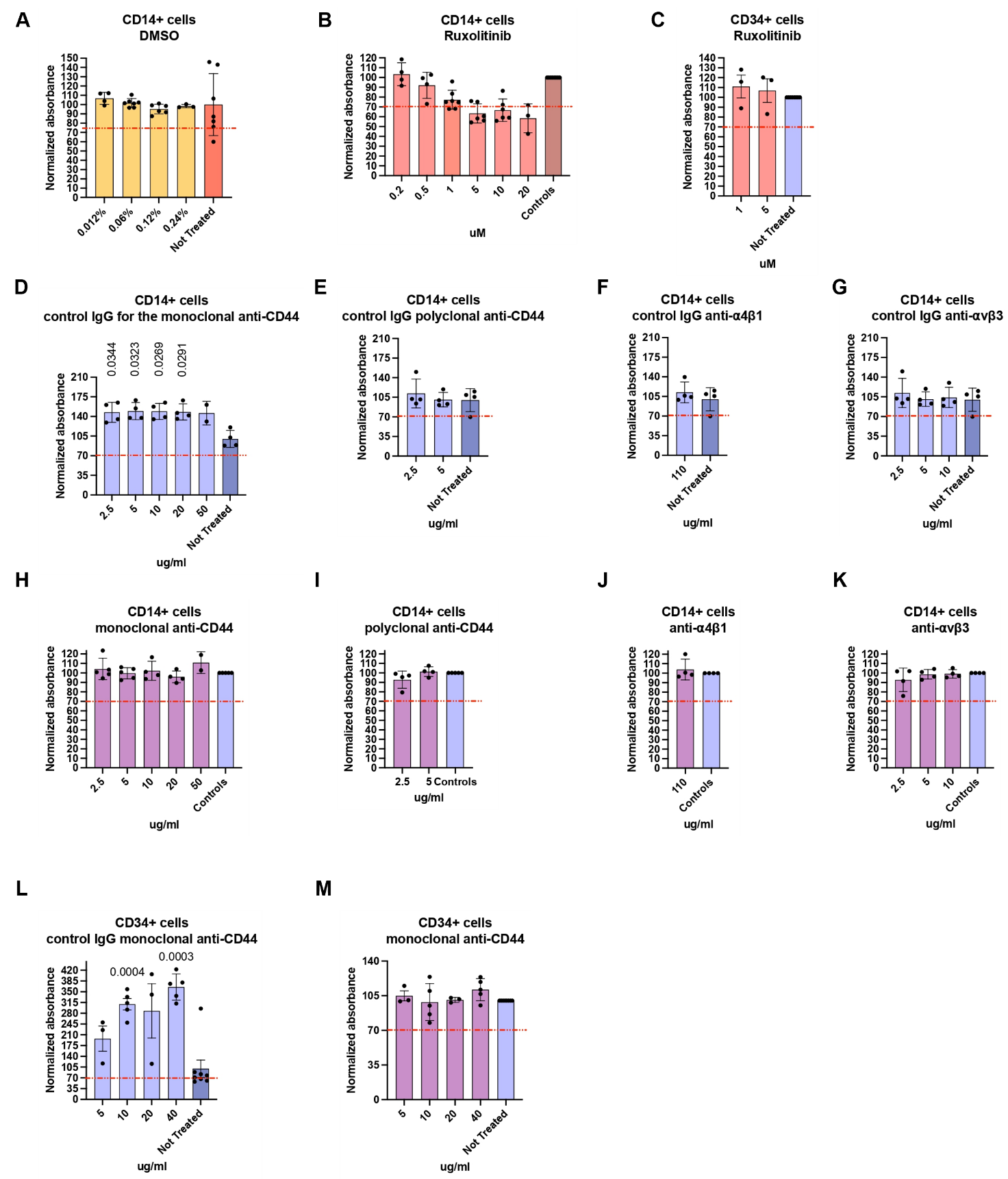
**

**Supplementary Figure 6. XTT assay results.** The red dashed line represents 70% viability. Panels A, B and C show XTT assay results evaluating cytotoxicity of Ruxolitinib and the relative control DMSO in HD CD14^+^ cells and HD CD34^+^ cells. Panels D, E, F and G display results of control IgG cytotoxicity evaluation in HD CD14^+^ cells as compared to not treated cells. Panels H, I , J and K show XTT assay results evaluating antibodies recognizing OPN receptors cytotoxicity in HD CD14^+^ cells as compared to control IgG samples at the same concentration. Panel L and M display XTT assay results for the monoclonal anti-CD44 antibody and corresponding control IgG in HD CD34^+^ cells. Comparisons between the treated samples and the control were performed by means of paired T-test. P-values are reported only if <0.05.

**Supplementary Figure 7**

**
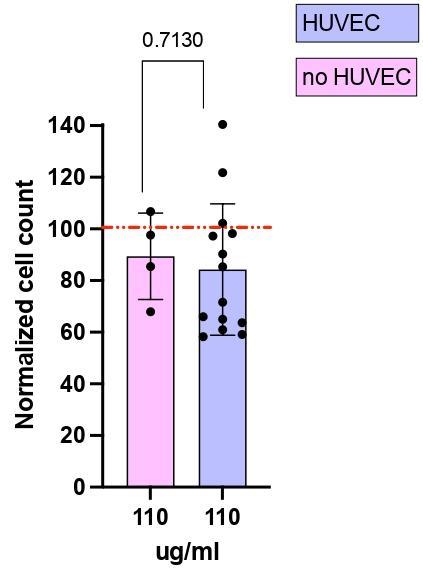
**

**Supplementary Figure 7: Migration results for the monoclonal anti-⍺4β1 antibodies with and without HUVEC on CD14+ cells.** Bar plot shows the effect on CD14^+^ cells migration due to the monoclonal anti-α4β1. Transwell samples with HUVEC are represented with purple bars while Transwell samples without HUVEC are illustrated with pink bars. The red dashed line represents 100% migration; the percentage of migration is normalized considering control IgG samples, not shown in this figure. Comparisons were performed by means of paired T-test.

**Supplementary Table 1**

**The list of MF patients included in the study with the corresponding clinical features. The figures where the patient sample was used is indicated.**

| **Patient** | **Age** | **Sex** | **Disease** | **Driver mutation** | **Treatment** | **Fig. 2b** | **Fig. 2e** | **Fig 3a, b** | **Fig 4e, f** | **Fig. 5a** | **Fig. 6a, b** | **Fig 6c** | **Fig 6d** | **Fig 6e** | **Fig 6h, i** | **Fig 6f, g** |
| --- | --- | --- | --- | --- | --- | --- | --- | --- | --- | --- | --- | --- | --- | --- | --- | --- |
| **Pt#1** | 71 | F | PMF | MPL W515L | androgen, HU, talidomide, Ruxo |  | **X** |  |  |  | **X** |  |  |  |  |  |
| **Pt#2** | 64 | M | pPV-MF | JAK2 V617F | No |  | **X** |  |  | **X** | **X** | **X** | **X** | **X** | **X** |  |
| **Pt#3** | 71 | M | PMF | JAK2 V617F | HU |  |  |  |  |  |  |  |  |  | **X** |  |
| **Pt#4** | 77 | F | PMF | CALR indel | HU, Ruxo |  |  |  |  |  | **X** |  |  |  |  |  |
| **Pt#5** | 75 | F | pET-MF | JAK2 V617F | HU, anagrelide |  |  |  |  |  | **X** |  |  | **X** | **X** |  |
| **Pt#6** | 60 | M | PMF | CALR indel | Ruxo | **X** |  |  | **X** |  | **X** |  |  |  |  |  |
| **Pt#7** | 78 | F | PMF | JAK2 V617F | Ruxo |  |  |  |  | **X** |  | **X** | **X** | **X** | **X** |  |
| **Pt#8** | 69 | F | PMF | JAK2 V617F | No |  |  |  |  |  |  |  | **X** |  |  |  |
| **Pt#9** | 63 | M | PMF | JAK2 V617F | No |  |  |  |  |  |  |  |  |  | **X** |  |
| **Pt#10** | 79 | M | pET-MF | CALR indel | Ruxo |  | **X** |  |  | **X** | **X** | **X** | **X** | **X** | **X** | **X** |
| **Pt#11** | 77 | F | PMF | JAK2 V617F | HU, anagrelide |  |  |  |  | **X** | **X** | **X** | **X** | **X** | **X** |  |
| **Pt#12** | 66 | F | PMF | JAK2 V617F | HU, Ruxo |  |  |  |  | **X** |  | **X** | **X** |  |  |  |
| **Pt#13** | 80 | F | PMF | JAK2 V617F | Danazol, EPO |  |  |  |  | **X** |  | **X** | **X** |  |  |  |
| **Pt#14** | 66 | M | pPV-MF | JAK2 V617F | No |  |  |  |  | **X** |  | **X** | **X** |  |  |  |
| **Pt#15** | 45 | F | pPV-MF | JAK2 V617F | HU |  |  |  |  | **X** |  | **X** | **X** |  |  |  |
| **Pt#16** | 71 | M | PMF | TN | HU |  |  |  |  | **X** |  | **X** | **X** |  | **X** |  |
| **Pt#17** | 54 | F | pPV-MF | JAK2 V617F | IFN, HU, Ruxo, 5-azacitidine |  |  |  |  | **X** |  | **X** | **X** |  |  |  |
| **Pt#18** | 55 | F | pPV-MF | JAK2 V617F | HU, anagrelide |  |  | **X** |  | **X** |  | **X** | **X** | **X** | **X** |  |
| **Pt#19** | 68 | M | pET-MF | JAK2 V617F | No |  |  |  |  | **X** |  | **X** | **X** |  |  |  |
| **Pt#20** | 80 | F | PMF | TN | HU |  |  |  |  |  |  |  |  | **X** | **X** |  |
| **Pt#21** | 67 | M | PMF | JAK2 V617F | HU |  |  | **X** |  | **X** |  | **X** | **X** | **X** | **X** |  |
| **Pt#22** | 62 | M | pPV-MF | JAK2 V617F | HU |  |  |  |  |  | **X** |  |  | **X** | **X** |  |
| **Pt#23** | 66 | F | pPV-MF | CALR indel | HU, Ruxo |  |  | **X** |  |  | **X** |  |  |  | **X** | **X** |
| **Pt#24** | 68 | M | pPV-MF | JAK2 V617F | HU |  |  |  |  |  |  |  |  |  |  | **X** |
| **Pt#25** | 73 | F | pPV-MF | JAK2 V617F | HU, Ruxo |  |  |  |  |  |  |  |  |  | **X** |  |
| **Pt#26** | 52 | M | PMF | JAK2 V617F | No |  |  | **X** |  |  |  |  |  | **X** | **X** |  |
| **Pt#27** | 45 | F | pPV-MF | JAK2 V617F | Cytoreduction |  |  | **X** |  |  |  |  |  |  |  |  |
| **Pt#28** | 43 | M | PMF | JAK2 V617F | Cytoreduction |  |  | **X** |  |  |  |  |  |  |  |  |
| **Pt#29** | 71 | M | pET-MF | JAK2 V617F | IFN, HU, anagrelide, Ruxo | **X** |  |  | **X** |  |  |  |  |  |  |  |
| **Pt#30** | 77 | M | pPV-MF | JAK2 V617F | HU, Ruxo | **X** |  | **X** | **X** |  |  |  |  |  |  |  |
| **Pt#31** | 53 | M | PMF | CALR indel | No |  |  | **X** |  |  |  |  |  |  |  |  |

Abbreviations: PMF: primary myelofibrosis, pPV-MF: post policythemia vera myelofibrosis; pET-MF: post essential thrombocythemia myelofibrosis; HU: hydroxyurea; Ruxo: Ruxolitinib; IFN: interferon; EPO: Erythropoietin
